# Arsenic-Induced PPARγ, with the Coordinated Action of p62, Inhibits Apoptosis and Necroptosis and Activates the DNA Damage Response in A549 Lung Cancer Cells, Leading to Carcinogenesis

**DOI:** 10.3390/cells15080659

**Published:** 2026-04-08

**Authors:** Hak-Ryul Kim, Seon-Hee Oh

**Affiliations:** 1 Division of Pulmonology, Department of Internal Medicine, Wonkwang University Hospital, Iksan 54538, Republic of Korea; kshryj@wonkwang.ac.kr; 2 School of Medicine, Chosun University, 309 Pilmundaero, Gwangju 61452, Republic of Korea

**Keywords:** mixed lineage kinase domain-like, necroptosis, PARP-1, p53, PPARγ, receptor-interacting protein 1, sodium arsenite

## Abstract

**Highlights:**

**What are the main findings?**

- Sodium arsenite activates PPARγ to promote DNA repair and inhibit apoptosis and necroptosis in NSCLC cells.
- Inhibiting PPARγ signaling induces apoptosis or necroptosis through PARP-1, MLKL, and p53.

**What are the implications of the main findings?**

- PPARγ is a key determinant of cell fate in arsenic-induced lung cancer.
- PPARγ represents a potential therapeutic target in arsenic-related carcinogenesis.

**Abstract:**

Arsenic exposure increases lung cancer risk, yet its molecular mechanisms remain unclear but are linked to peroxisome proliferator-activated receptor gamma (PPARγ). We investigated PPARγ-related molecules affected by sodium arsenite (NaAR) in non-small cell lung cancer (NSCLC) cells using immunochemical, gene knockdown, and immunoprecipitation approaches. PPARγ was critical for NSCLC growth, as high PPARγ-expressing A549 cells proliferated more than low-expressing H1299 cells after NaAR treatment. In A549 cells, NaAR upregulated polyubiquitinated PPARγ, activating cell cycle arrest and DNA damage response pathways. Rather than inducing significant caspase-dependent apoptosis, NaAR activated nuclear factor-kappa B and downregulated mixed lineage kinase domain-like (MLKL) via K63-linked polyubiquitinated receptor-interacting protein kinase 1, thereby inhibiting apoptosis and necroptosis. PPARγ knockdown or NAD^+^ supplementation induced PARP-1 hyperactivation and MLKL upregulation, leading to DNA damage and necroptosis. PARP-1 inhibition by 3-aminobenzamide induced apoptosis, indicating that PPARγ regulates apoptosis and necroptosis through PARP-1 activation. Proteasome inhibition increased polyubiquitinated PPARγ but not p53. Leptomycin B induced PPARγ degradation and p53 accumulation, promoting necroptosis and apoptosis, suggesting cytoplasmic p53 contributes to cell death. p62 interacted with PPARγ and p53, and its knockdown suppressed their NaAR-induced upregulation. In conclusion, NaAR-induced PPARγ promotes A549 cell survival by enhancing DNA repair and inhibiting apoptosis and necroptosis via cooperation with p53 and p62, highlighting PPARγ as a potential therapeutic target.

## 1. Introduction

Lung cancer, which accounts for more than 30% of cancer-related deaths worldwide [1], is primarily caused by smoking. However, epidemiological studies have shown environmental pollution to be an equally important risk factor, with air pollution due to industrial development as well as contaminated crops and drinking water contributing to an increase in the disease incidence in non-smokers worldwide [2]. Arsenic, which originates from both anthropogenic and natural sources, is a major air and soil contaminant. Heavy exposure to this toxic metalloid via inhalation and ingestion can significantly increase the risk of developing serious diseases [3]. Epidemiological studies have established a positive correlation between arsenic exposure and lung cancer risk [4], and the International Agency for Research on Cancer classifies arsenic as a human carcinogen [5]. Moreover, arsenic intake via the inhalation of contaminated dust and air in commercial areas has been shown to be higher than that from contaminated food [6].

Peroxisome proliferator-activated receptors (PPARs), which are ligand-dependent transcription factors belonging to the nuclear hormone receptor family, are composed of three isoforms: α, γ, and δ [7]. In addition to playing essential roles in adipogenesis and glucose homeostasis [8], PPARγ is also expressed in various types of cancer, such as in non-small cell lung cancer (NSCLC), where its expression correlates positively to the histological type and grade [9,10], making it a potential therapeutic target [11,12]. Despite its involvement in carcinogenesis, PPARγ also exerts anticancer effects by regulating cell proliferation, apoptosis, and inflammation. It inhibits the proliferation of lung cancer cells by regulating growth arrest and DNA damage-inducible gene 153 (GADD153) [13], extracellular signal-regulated kinase (ERK) [14], death receptor 5 (DR5) [15], and p21 [16]. Moreover, it induces reactive oxygen species-mediated apoptosis [17]. Furthermore, in human lung adenocarcinoma cells, PPARγ enhanced TRAIL-mediated autophagic cell death [18]. Thus, decreased PPARγ expression may be associated with a poor prognosis in lung cancer. Conversely, PPAR activation and the ensuing effects on anti-apoptotic or oxidative defense mechanisms have been implicated in the development of cancer treatment resistance [19]. Therefore, PPAR inhibition may be a promising strategy for overcoming therapeutic resistance. However, the exact role of PPARγ in lung carcinogenesis remains unclear.

The cargo receptor p62, also known as sequestosome-1 (SQSTM1), is involved in cell quality control via the ubiquitin-proteasome system and autophagy [20]. It also functions as a molecular hub for various metabolic pathways, apoptosis, and carcinogenesis [21]. p62 has two nuclear localization signals and a nuclear export signal that facilitate its dynamic translocation between the nuclear and cytoplasmic compartments, where it regulates protein quality control [22]. Although primarily localized in the cytoplasm, p62 exists in nuclear compartments such as promyelocytic leukemia (PML) bodies and is involved in the degradation of ubiquitinated nuclear proteins [23,24]. Cytoplasmic p62 delivers damaged or unwanted cellular components and ubiquitinated targets to the autophagosomes or proteasomes [20]. In addition to being an autophagy adapter, p62 is overexpressed in many types of tumors, where it acts as either a tumor suppressor or promoter [25]. It contributes to cell proliferation and tumorigenesis by activating the Nrf2–heme oxygenase-1 (HO-1) antioxidative pathway [26]. It is also involved in cell cycle progression through its interaction with cyclin-dependent kinase 1 (CDK1) [27]. Given that PPARγ also modulates the cell cycle through the downregulation of CDK1 [28], it is possible that it interacts with p62 in regulating cell cycle progression and carcinogenesis. Indeed, p62 promotes PPARγ transcriptional activity by enhancing its interaction with retinoid X receptor, which in turn promotes autophagy [29]. However, the pathophysiological roles of p62 and PPARγ remain unclear.

In this study, we investigated PPARγ expression by p53-expressing A549 lung cancer cells in response to sodium arsenite (NaAR) exposure and the molecular mechanisms underlying NaAR-induced carcinogenesis. Our findings suggest that PPARγ may be a potential therapeutic target for arsenic-induced carcinogenesis, at least in lung cancer cells expressing p53.

## 2. Materials and Methods

### 2.1. Reagents and Antibodies

Sodium arsenite (NaAsO_2_), 3-Aminobenzamide (A0788), NAD^+^ (481911), 3-(4,5-dimethylthiazol-2-yl)-2,5-diphenyltetrazolium bromide (MTT; M2128), MG132 (M8699), Hoechst 33,342 (B2261), anti-β-actin antibody (ab-8226), and neutral-buffered formalin (HT5011) were obtained from Sigma-Aldrich (St. Louis, MO, USA). SQSTM1/p62 (sc-25575), Fas (sc-7886), p53 (sc-126), RIP3 (sc-374639), p21 (sc-6246), and K48-ubiquitin (sc-8017), as well as rhodamine-conjugated goat anti-rabbit (sc-2091) and FITC-conjugated goat anti-mouse (sc-2010) secondary antibodies, were purchased from Santa Cruz Biotechnology (Santa Cruz, CA, USA). PARP-1 (#9532), procaspase-3 (#9662), cleaved caspase-3 (#9661), phospho-p53 (S15, #9284), phospho-ATR (#2853), ATR (#2790), phospho-Chk1 (#2348), Chk1 (#2360), MLKL (#26539), p21 (#73543), K63-polyubiquitin (#12930), RIP1 (#3493), phospho-γ-H2AX (S139, #2577), PPARγ (2443), and poly/mono-ADP-ribose (#89190) antibodies were purchased from Cell Signaling (Beverly, MA, USA). SQSTM1/p62 (H00008878-M01) antibodies were purchased from Abnova (Taipei, Taiwan).

### 2.2. Cell Culture

Human lung cancer cell lines A549 (CCL-185^TM^), H1299 (CRL-1803^TM^), and H460 (HTB-177^TM^) were obtained from the American Type Culture Collection (ATCC, Rockville, MD, USA). Cells were cultured in Dulbecco’s modified Eagle’s medium (DMEM; WelGene, Gyeongsan, South Korea), supplemented with 10% fetal bovine serum (WelGene, S001) and penicillin-streptomycin (WelGene, LS203). Cultures were maintained at 37 °C in a humidified atmosphere containing 5% CO_2_. All cell lines were routinely tested and confirmed to be free of mycoplasma contamination and were used at passages 3–12.

### 2.3. Cytotoxicity Assays

Cell viability was assessed using the MTT assay. Cells were seeded in 48-well plates at a density of 1 × 10^5^ cells/mL (200~250 μL per well) and cultured for 48 h. Following treatment, MTT was added to a final concentration of 0.5 mg/mL, and the cells were incubated at 37 °C for 2 h. The resulting formazan crystals were dissolved in dimethyl sulfoxide (DMSO), and absorbance was measured at 540 nm using a microplate reader (Perkin-Elmer, Waltham, MA, USA). All experiments were performed at least three times independently. Data are presented as mean ± standard deviation (SD), expressed as fold change relative to the control.

### 2.4. Transfection

Cells were plated in 6-well plates at a density of 5 × 10^5^ cells per well and transfected with 100 pmol of siRNA using Lipofectamine^TM^ RNAiMAX reagent (Invitrogen, Carlsbad, CA, USA) in accordance with the manufacturer’s protocol. After 6 h, the medium was replaced with fresh complete medium, and cells were further incubated for 24 h prior to subsequent treatment. The siRNA sequences used were as follows: PPARγ, 5′-GACAAAUCACCAUUCGUAUU-3′; p62, 5′-CUUGUAGUUGCAUCACGUA-3′; and p53, 5′-GUCUGUUAUGUGCACGUAC-3′ (Bioneer, Daejeon, South Korea). A non-targeting control siRNA was obtained from Sigma-Aldrich (St. Louis, MO, USA; SIC001).

### 2.5. Immunoblotting and Immunoprecipitation

Cells were lysed in lysis buffer (#9803; Cell Signaling Technology) containing a protease cocktail (#04693132001; Roche, Basel, Switzerland). The proteins (15–35 μg) were resolved using 10–12% SDS-PAGE and transferred to polyvinylidene difluoride membranes (IPVH00010; Millipore, Burlington, MA, USA). After blocking the membranes with 5% skim milk, they were probed with primary and secondary antibodies. The proteins were then visualized using a chemiluminescent substrate (WBKLS0100; Millipore). For immunoprecipitation, cells were lysed in lysis buffer (0.05 M Tris-HCl (pH 7.4), 250 mM NaCl, 0.25% Triton X-100, 10% glycerol) containing a protease cocktail. Total proteins (800 μg) were pre-cleared using 50% Protein G Plus-Agarose beads (sc-2002; Santa Cruz) and centrifuged at 12,000× *g* for 10 min at 4 °C. The supernatants were incubated overnight with p62/SQSTM1 and mouse IgG antibodies (#12-371; Sigma-Aldrich) at 4 °C. The immunocomplexes were captured using Protein G Plus-Agarose beads and washed several times with ice-cold PBS. The washed beads were resuspended in 2× Laemmli loading buffer and then boiled, and the proteins were eluted and processed for immunoblotting.

### 2.6. Immunofluorescence

Cells grown on coverslips (D111580; Marienfeld, Lauda-Königshofen, Germany) were fixed with ice-cold neutral-buffered formalin for 10 min. After fixation, cells were rinsed with PBS and permeabilized using 0.05% Triton X-100 (#T8787; Sigma) for 20 min. Following additional PBS washes, non-specific binding was blocked with 2% BSA (ALB001; Bioshop, Burlington, ON, Canada). The cells were then incubated with primary antibodies targeting p62, p53, and PPARγ, followed by appropriate fluorescently labeled secondary antibodies. Nuclei were stained with Hoechst 33342 (1 μg/mL), and fluorescence images were obtained using a Nikon Eclipse TE300 microscope.

### 2.7. Statistical Analysis

All experiments were conducted in at least three independent replicates. Results are expressed as mean ± standard deviation (SD). Statistical comparisons among groups were performed using one-way analysis of variance (ANOVA), and differences were considered significant when the *p*-value was <0.05.

## 3. Results

### 3.1. PPARγ Expression Was Inversely Correlated with Cell Growth in NaAR-Exposed Human Non-Small Cell Lung Cancer Cells

**Figure 1:**
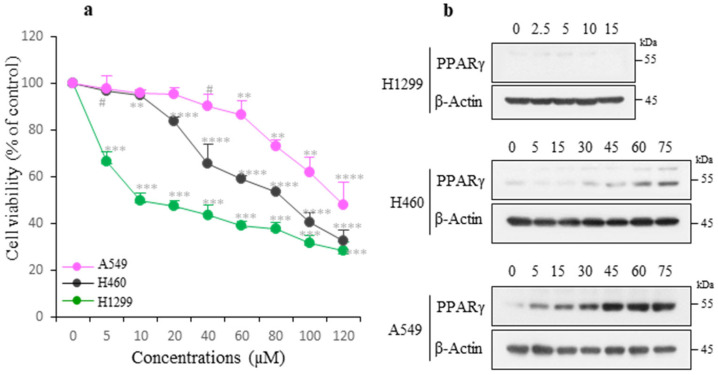
Viability of different NSCLC cell lines and PPARγ expression in response to NaAR. (**a**) Cells were treated with increasing concentrations of NaAR for 24 h, and cell viability was determined using the MTT assay. Data are expressed as the mean ± SD of fold increase compared with the untreated control from three independent experiments. ^#^ *p* < 0.05, ** *p* < 0.0002, *** *p* < 0.00002, **** *p* < 0.000002. (**b**) Cells were treated with NaAR as described in part (**a**), harvested, and lysed. The PPARγ expression levels were assessed using immunoblotting, with β-actin as the loading control. Data shown are based on over three separate experiments.

To elucidate the sensitivity of NSCLC cells to NaAR, three lung cancer cell lines were treated with increasing concentrations of NaAR for 24 h, and their proliferation was analyzed using the MTT assay. The viability of H1299 (p53^−/−^), H460 (p53^+/+^), and A549 (p53^+/+^) cells decreased in a dose-dependent manner, with IC_50_ values of 28, 62, and 118 μM NaAR, respectively. The H1299 cells were the most sensitive to NaAR, followed by the H460 and A549 cells (Figure 1a). To investigate whether PPARγ is involved in the inhibition of lung cancer cell proliferation, we analyzed its protein levels in the three NaAR-exposed cell lines. The H1299 cells did not express significant levels of PPARγ, whereas the H460 cells showed significant PPARγ expression at NaAR concentrations of 60 µM or more. By contrast, the A549 cells, which were the least sensitive to NaAR, expressed high levels of PPARγ (Figure 1b). These results suggest that PPARγ may be an important regulator in determining sensitivity to NaAR.

### 3.2. NaAR Affected Apoptosis and Cell Cycle Progression in A549 and H1299 Cells

**Figure 2:**
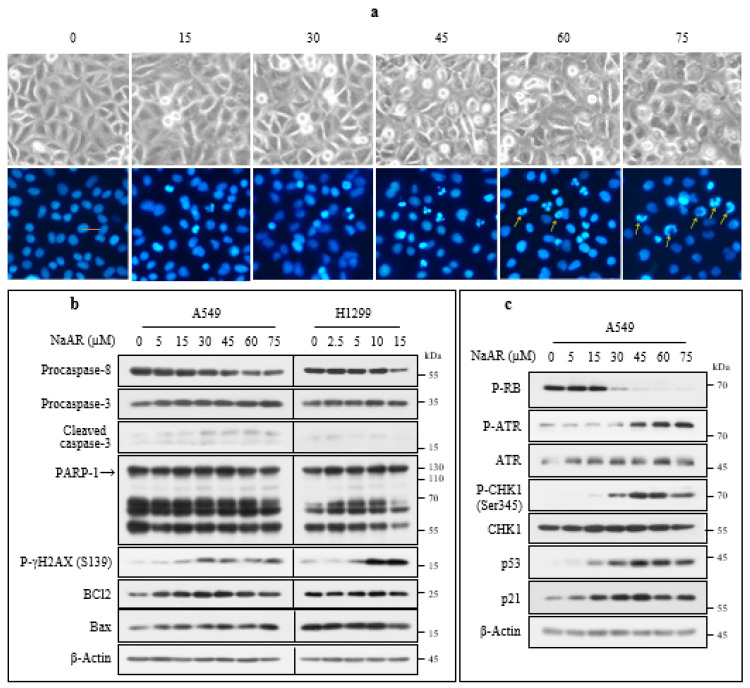
Expression of proteins associated with apoptosis and cell cycle regulation in NaAR-exposed A549 cells. (**a**) A549 cells were exposed to increasing concentrations of NaAR for 18 h, and morphological alterations were examined by phase-contrast microscopy. Nuclear staining was performed using Hoechst 33342, and fluorescence images were captured with Nikon Eclipse TE300 microscope (200×). (**b**) A549 and H1299 cells were treated with the indicated concentrations of NaAR for 18 h, followed by cell collection and lysis. Protein expression levels were analyzed by immunoblotting. (**c**) A549 cells were treated under the same conditions, and cell lysates were subjected to immunoblot analysis to evaluate target protein expression, β-actin used as a loading control. Arrows indicate non-apoptotic chromatin changes. Data represent results from at least three independent experiments. The arrows indicate large-scale segmented chromatin.

Because the A549 and H1299 cells showed the biggest difference in PPARγ expression levels, we compared their morphologies and the signaling pathways involved in their sensitivity to NaAR. The A549 cells showed different morphological changes depending on the degree of NaAR exposure. Below the IC50 value, the number of round and floating cells increased with increasing NaAR concentration; on the other hand, in cells treated with high concentrations, the number of floating cells decreased significantly and turned into oval cells (Figure 2a). Hochest 33342 staining revealed typical apoptotic features such as condensed and fragmented nuclei in the 15 μM NaAR-treated cells, and their numbers increased in the presence of up to 45 μM NaAR. However, in cells treated with ≥60 μM of NaAR, segmented chromatin structures along the nuclear membrane were observed (Figure 2a, arrows). Most of the H1299 cells exposed to 10 and 15 µM NaAR were round and floated in the medium (Supplementary Figure S1).

To study the molecular mechanisms underlying NaAR-induced growth inhibition in A549 cells, we first examined their expression of apoptosis-related proteins in the presence of varying concentrations of NaAR. The cellular procaspase-8 levels decreased significantly in a dose-dependent manner, whereas only low levels of caspase-3 and poly-(ADP ribose) polymerase-1 (PARP-1) cleavage were induced. A significantly lower level of Ser139-phosphorylated histone H2AX (p-γH2AX), a marker of DNA double-strand breaks, was induced in the A549 cells compared with that in the H1299 cells. However, the changes in caspase levels were similar in both types of NaAR-exposed cells. Additionally, both the anti-apoptotic B-cell lymphoma 2 (BCL2) and pro-apoptotic BCL2-associated X apoptosis regulator (Bax) proteins were significantly increased in the A549 cells after NaAR exposure, whereas no significant changes in these molecules were evident in the H1299 cells (Figure 2b). Next, we examined proteins involved in cell cycle progression to determine if PPARγ expression is associated with A549 cell proliferation. Phosphorylated retinoblastoma-associated protein (p-RB), a cell proliferation marker, began decreasing at NaAR concentrations above 30 µM. Phosphorylated ataxia telangiectasia-mutated and Rad3-related (p-ATR) kinase and its downstream targets (phosphorylated checkpoint kinase 1 (p-CHK1), p53, and p21) were upregulated in a dose- and time-dependent manner (Figure 2c; Supplementary Figure S2). By contrast, p-ATR was upregulated in NaAR-exposed H1299 cells, but its downstream targets were not (Supplementary Figure S3). Although both cell lines were damaged by NaAR, they were insensitive to canonical caspase-dependent apoptosis, suggesting that the low sensitivity of A549 cells to the toxic compound may be due to induction of the DNA damage response and inhibition of apoptosis.

### 3.3. NaAR Inhibited DNA Damage and Apoptosis by Regulating the PARP-1 Activation Level

**Figure 3:**
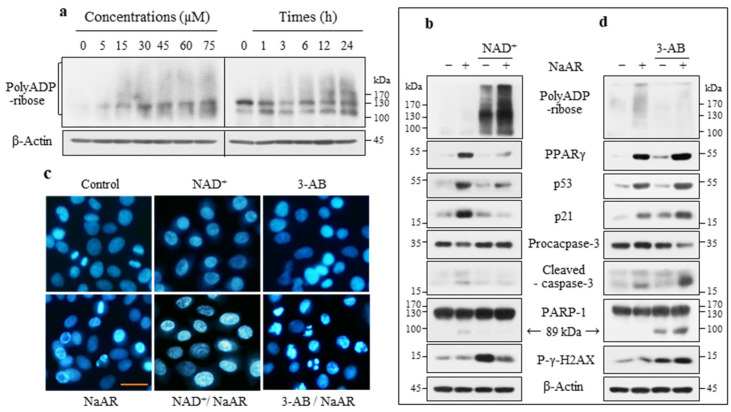
NaAR-induced cell death and DNA damage is dependent on PARP-1 activation. (**a**) A549 cells were treated with increasing NaAR concentrations for 18 h or with 65 µM NaAR for up to 24 h. Target proteins in the cell lysates were then evaluated using immunoblotting. (**b**) Immunoblot of target proteins from A549 cells exposed to 65 µM NaAR for 18 h, with or without 50 µM NAD+ pretreatment for 2 h. (**c**) Hoechst 33342-stained cells. Lung cancer cells cultured on coverslips were treated with 65 µM NaAR for 18 h, with or without 50 µM NAD+ or 10 μM 3-AB pretreatment for 2 h, respectively. Scale bar: 25 µm. (**d**) Immunoblot of target proteins from A549 cells exposed to 65 µM NaAR for 18 h, with or without 10 μM 3-AB pretreatment for 2 h. β-Actin was used as the loading control.

Because PARP-1 cleavage was not significantly induced in A549 cells in response to NaAR, even at high concentrations (Figure 2b), we investigated whether NaAR induces PARP-1 activation. Compared with the H1299 cells, the A549 cells showed a much lower level of PARP-1 polyADP-ribosylation (Figure 3a and Figure S3). Subsequently, we used NAD+, an activated PARP-1 substrate, to examine the role of PARP-1 activation in NaAR-induced growth inhibition. NAD+ supplementation greatly increased the level of NaAR-induced polyADP-ribosylation, resulting in decreased PPARγ, p53, and p21 levels and the inhibition of caspase-3 and PARP-1 cleavage. Additionally, p-γH2AX, which was significantly upregulated in cells treated with NAD+ alone, was reduced by NAD+ and NaAR co-treatment, albeit the level was still higher than that under NaAR alone (Figure 3b).

NaAR exposure induced different chromatin changes, including condensation and fragmentation, granular and condensed morphology, and condensation into rings along the nuclear membrane. Most of the cells exposed to NAD+ contained granular and condensed chromatin (Figure 3c). Next, we investigated the effect of 3-aminobenzamide (3-AB; a PARP-1 inhibitor) on NaAR-induced growth inhibition. 3-AB almost completely inhibited NaAR-induced polyADP-ribosylation, resulting in increased PPARγ, p53, p21, and p-γH2AX levels and marked caspase-3 and PARP-1 cleavage (Figure 3d). Consistent with these results, 3-AB treatment induced apoptotic nuclear changes in several cell lines (Figure 3c). Collectively, these results indicate that NaAR-mediated cytoprotection requires PARP-1 activation rather than hyperactivation.

### 3.4. NaAR Induced RIP1-Mediated NF-κB Activation and Inhibited Apoptosis and Necroptosis

**Figure 4:**
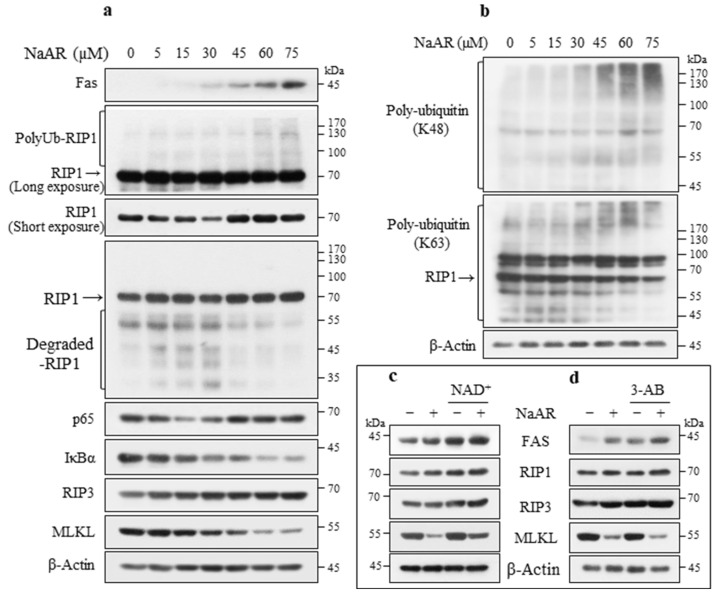
NaAR exposure induces K63-linked RIP1-mediated NF-κB activation and MLKL downregulation. (**a**,**b**) Immunoblot of target proteins from A549 cells treated with increasing concentrations of NaAR for 18 h. (**c**) Immunoblot of target proteins from A549 cells exposed to 65 µM NaAR for 18 h, with or without 50 µM NAD^+^ pretreatment for 2 h. (**d**) Immunoblot of target proteins from A549 cells exposed to 65 µM NaAR for 18 h, with or without 10 μM 3-AB pretreatment for 2 h. β-Actin was used as the loading control.

Given that NaAR exposure reduced procaspase-8 but did not induce significant procaspase-3 and PARP-1 cleavage in A549 cells (Figure 2b), indicating insensitivity to caspase-dependent apoptosis, we investigated the involvement of caspase-independent cell death pathways. NaAR upregulated the expression levels of Fas (CD95/APO-1) and receptor-interacting protein kinase 1 (RIP1; a pivotal regulator of cell survival and death). However, RIP1 showed a biphasic expression pattern of gradual degradation up to 30 μM NaAR followed by a concentration-dependent increase in ubiquitination, indicating that its kinase activity was inhibited at low NaAR concentrations. The post-transcriptional modification of RIP1 by K63-linked ubiquitination plays a critical role in cell survival signaling through nuclear factor-kappa B (NF-κB) activation [30]. The K63- and K48-linked ubiquitinated (Ub) proteins began to increase at NaAR concentrations above 30 μM, with the former peaking at 60 μM and then decreasing slightly (Figure 4b), indicating that high concentrations of NaAR induce Fas-mediated K63-linked RIP1 activation. Furthermore, the NF-κB p65 levels showed a similar pattern to that of the RIP1 levels, whereas phosphorylated inhibitor of kappa B alpha (p-IKα) gradually decreased. These results indicate that RIP1 is recruited to Fas signaling and promotes NF-κB activation through K63-linked ubiquitination for cell survival. Conversely, no significant levels of K63- and K48-linked Ub-proteins were induced in the NaAR-exposed H1299 cells (Supplementary Figure S4). We next examined whether RIP1 is involved in ripoptosis via complex IIa (RIP1–Fas-associated death domain (FADD)–caspase-8) or necroptosis via complex IIb (RIP1–RIP3–MLKL) formation. Although NaAR-mediated RIP1 upregulation failed to induce caspase-3 cleavage, it may be involved in ripoptosis through riposome (complex IIa) formation, because caspase-8 was downregulated after NaAR exposure. Furthermore, NaAR exposure upregulated RIP3 but failed to induce necrosome (complex IIb) formation, owing to MLKL degradation. These results suggest that NaAR inhibits Fas-mediated apoptosis and necroptosis. Consistently, NaAR induced the expression of survival signaling molecules such as p-AKT and p-ERK. AKT inhibition abolished NaAR-induced p-ERK and induced significant PARP-1 cleavage (Supplementary Figure S5a,b). Therefore, the low sensitivity of A549 cells to NaAR may be due to their inability to competently induce apoptosis and necroptosis. Consistent with these results, NAD+ supplementation significantly induced PARP-1 hyperactivation (Figure 3a), resulting in the upregulation of Fas, RIP1, RIP3, and MLKL (which was downregulated by NaAR) (Figure 4c). Conversely, 3-AB treatment resulted in significant caspase-3 and PARP-1 cleavage (Figure 3d), RIP1 and RIP3 upregulation, and a further decrease in MLKL expression (Figure 4d), indicating that PARP-1 inactivation can lead to ripoptosis. Taken together, these results suggest that NaAR exposure switches cell death modes by regulating PARP-1 activation and MLKL expression.

### 3.5. PPARγ Inhibited PARP-1 Hyperactivation and Necroptosis

**Figure 5:**
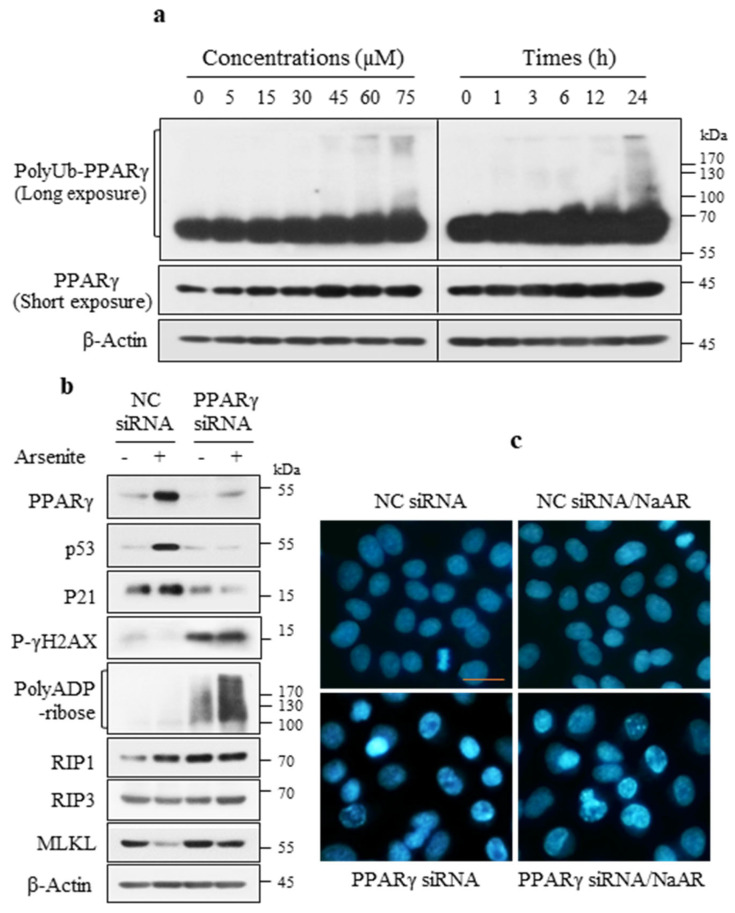
NaAR induces polyUb-PPARγ, and PPARγ knockdown leads to PARP-1 hyperactivation and necroptosis. (**a**) Immunoblot of PPARγ proteins from A549 cells treated with increasing NaAR concentrations for 18 h or with 65 µM NaAR for up to 24 h. (**b**) Immunoblot of target proteins from cells transfected with negative control (NC) or PPARγ siRNAs and then exposed to 65 µM NaAR for 18 h. (**c**) Cells were transfected with NC or PPARγ siRNAs and then exposed to NaAR as described in b, and the nuclei were stained with Hoechst 33342. Images were acquired with a fluorescence microscope. Scale bar: 25 µm. β-Actin was used as the loading control.

PolyUb- and monomer-PPARγ were induced in the NaAR-exposed A549 cells in a dose- and time-dependent manner (Figure 5a). Therefore, we investigated the role of PPARγ in NaAR-induced signaling pathways. siRNA-mediated *PPARγ* knockdown decreased the NaAR-induced upregulation of p53 and p21, increased p-γH2AX, promoted PARP-1 hyperactivation, and upregulated RIP1, RIP3, and MLKL expression (Figure 5b), indicating that PPARγ suppression causes extensive DNA damage and subsequent PARP-1 hyper-activation and necroptosis. Morphological analysis revealed that *PPARγ* knockdown significantly increased the number of nuclei with granular chromatin (large-scale DNA fragmentation) alterations (Figure 5c). Consistent with this, *p53* knockdown also reduced NaAR-induced PPARγ upregulation, which promoted the subsequent molecular effects described above (Supplementary Figure S6). These results indicate that PPARγ inhibits DNA damage-mediated PARP-1 hyper-activation and necroptosis.

### 3.6. PPARγ Stability Was Regulated by Proteasomes

**Figure 6:**
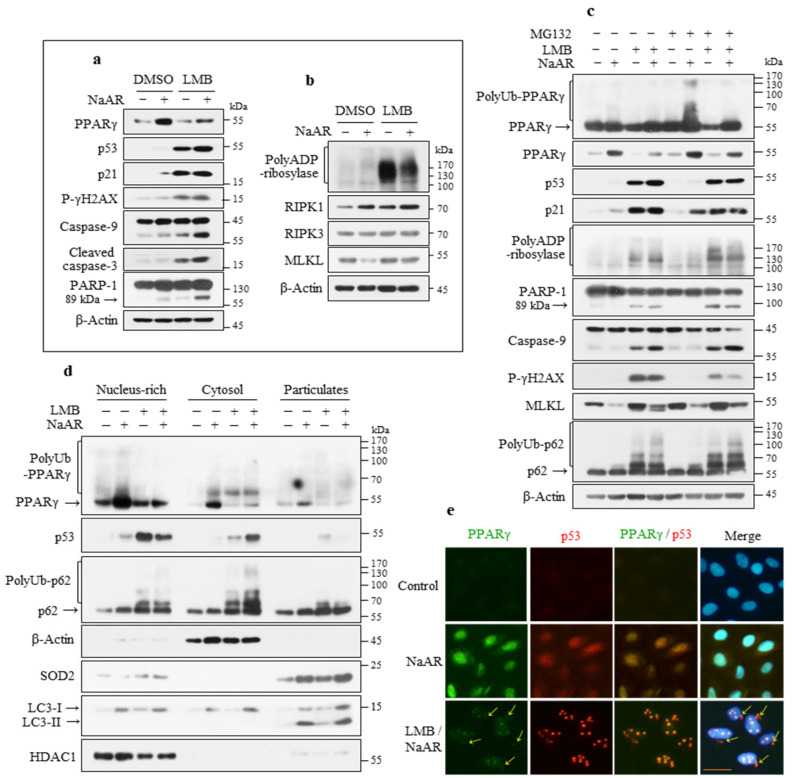
The stability of PPARγ in NaAR-exposed A549 cells is regulated by proteasomes. (**a**,**b**) Immunoblot of target proteins from A549 cells exposed to 65 µM NaAR for 18 h, with 25 nM LMB or DMSO pretreatment for 2 h. (**c**) Immunoblot of target proteins from cells treated with 65 µM NaAR for 18 h in the presence of 25 nM LMB or 5 µM MG132 or both inhibitors. β-Actin was used as the loading control. (**d**) Immunoblot of target proteins from cells treated as described in (**a**) and (**b**) and subjected to subcellular fractionation into nucleus-rich, cytosolic, and particulate fractions. The purities of the nuclear, autophagosome, mitochondrial, and cytosolic fractions were determined by immunoblotting for HDAC1, LC3-II, SOD2, and β-actin, respectively (n = 3). (**e**) Cells on coverslips were treated as in (**a**,**b**), fixed, and stained with PPARγ (green) and p53 (red), followed by FITC- and rhodamine-conjugated secondary antibodies. The arrows indicate p53 in the cytosol. Nuclei were counterstained with Hoechst 33342 (blue) and images were acquired by fluorescence microscopy. Arrows indicate apoptotic nuclei. Scale bar: 25 µm.

To further investigate the role of PPARγ in the NaAR-induced signaling pathways, cells were treated with leptomycin B (LMB), which inhibits the nuclear export signal of PPARγ. LMB treatment led to significant p53, p21, and p-γH2AX accumulation, but markedly decreased the PPARγ level, resulting in apoptosis through increased caspase-9 and caspase-3 activation and subsequent PARP-1 cleavage (Figure 6a). Moreover, LMB treatment substantially induced PARP-1 hyperactivation and upregulated RIPK1, RIPK3, and MLKL expression (Figure 6b). These results suggest that, unlike p53, PPARγ does not appear to be LMB-dependent and its decrease and p53 increase can promote necroptosis and caspase-dependent apoptosis.

To elucidate whether PPARγ protein stability is dependent on proteasome activity, A549 cells were treated with MG132 (a proteasome inhibitor) with or without NaAR. MG132 treatment upregulated the levels of mouse double minute 2 homolog (MDM2; also known as E3 ubiquitin ligase) and p21 relative to those in the control and NaAR-exposed groups. MG132 also significantly upregulated the polyUb- and monomer-PPARγ levels, but not the p53 level, indicating that PPARγ protein stability may be regulated by proteasome activity (Supplementary Figure S7). Based on these results, we investigated whether the LMB-induced decrease in PPARγ and increase in p53 levels were affected by the proteasome inhibitor by treating cells with NaAR in the presence of either LMB or MG132 or both (Figure 6c). As previously found, MG132 alone resulted in further NaAR-induced monomer- and polyUb-PPARγ accumulation, whereas LMB+MG132 co-treatment slightly reversed the LMB-induced downregulation of these proteins, indicating that PPARγ protein stability is regulated by nuclear proteasomes. Treatment with MG132 with or without NaAR did not significantly increase p53 levels compared with LMB-alone or LMB+MG132 treatments, whereas LMB+MG132 co-treatment resulted in similar p53 and p21 expression levels to those of LMB treatment alone, indicating that p53 protein stability is not affected by proteasome activity. Similar to LMB+MG132, LMB alone induced PARP-1 hyperactivation, PARP-1 cleavage, and caspase-9 activation. Unlike MG132, LMB significantly induced p-γH2AX and MLKL expression, but this effect was counteracted by co-treatment with MG132. Additionally, polyUb-p62 levels were markedly increased in both the LMB-alone and LMB+MG132 groups.

Next, we examined the subcellular localization of PPARγ degradation using fractionation analysis. Cells were separated into insoluble (nucleus-enriched membrane), soluble (cytosolic), and particulate (including mitochondria, autophagosomes, and endoplasmic reticulum) fractions. The purity of each fraction was verified by immunoblotting with specific marker proteins (Figure 6d).

Nuclear PPARγ translocated to the cytosolic and particulate fractions upon NaAR exposure and was degraded following LMB treatment in all three compartments. Unexpectedly, p53 accumulated mainly in the nucleus after LMB-alone treatment but was distributed equally in both the nuclear and cytoplasmic compartments after LMB+NaAR co-treatment. Additionally, polyUb-p62 accumulated in all three compartments, particularly the cytoplasm, and showed a localization pattern similar to that of p53. These results indicate that PPARγ is degraded in the nucleus, and p53 is partly independent of chromosome region maintenance 1 (CRM1). Indeed, cells co-treated with MG132+NaAR or LMB+MG132+NaAR showed an increase in nuclear PPARγ, suggesting that PPARγ may be targeted for degradation by the nuclear proteasome system (Supplementary Figure S8).

We also examined the subcellular locations of PPARγ and p53 after LMB treatment using immunofluorescence staining (Figure 6e). In control cells, the PPARγ and p53 staining intensities were minimal. After NaAR exposure, both proteins were homogeneously distributed in the nucleus with strong fluorescence signals but showed weak staining in the cytoplasm. However, with NaAR+LMB co-treatment, PPARγ appeared as faint globular spots in the nucleus, whereas p53 appeared as distinct and strong globular spots with notable fluorescence intensity in both the nucleus and cytoplasm. Furthermore, although LMB treatment led to PPARγ degradation, the protein retained its ability to co-localize with p53 in both the nucleus and cytoplasm. Taken together, these data suggest that the stability of PPARγ and p53 is regulated by different mechanisms.

### 3.7. p62 Stabilized p53 to Induce Apoptosis and Inhibit Necroptosis

**Figure 7:**
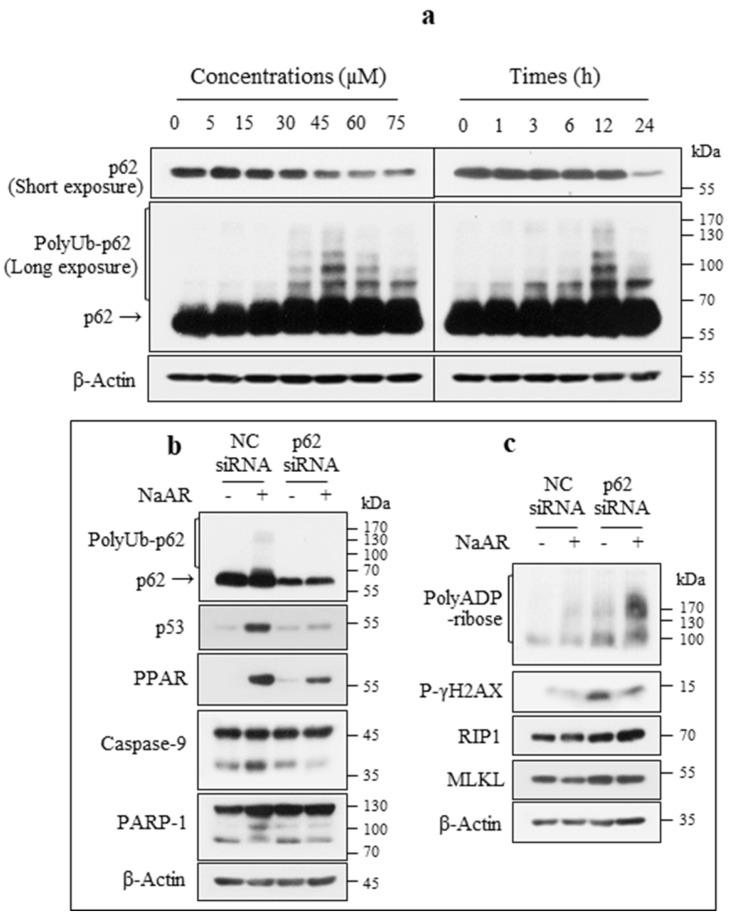
p62 regulates p53 stability in NaAR-exposed A549 cells. (**a**) Immunoblot of p62 proteins from A549 cells treated with increasing concentrations of NaAR for 18 h or with 65 µM NaAR for up to 24 h. (**b**,**c**) Immunoblot of target proteins from cells transfected with negative control (NC) or p62 siRNAs and then exposed to 65 μM NaAR for 18 h. β-Actin was used as the loading control (n = 3).

The subcellular location of p62 was similar to that of p53 in the NaAR-exposed A549 cells (Figure 6d). To investigate the involvement of p62 in p53 protein stability, we examined its expression in NaAR-exposed A549 cells. These cells expressed monomer- and polyUb-p62. Monomer-p62 gradually decreased in a dose- and time-dependent manner, whereas polyUb-p62 peaked at 45 μM NaAR and 12 h of incubation and then gradually decreased (Figure 7a). *p62* knockdown decreased NaAR-induced p53 and PPARγ upregulation as well as caspase-9 and PARP-1 cleavage (Figure 7b) but induced PARP-1 hyperactivation and p-γH2AX, RIP1, RIP3, and MLKL expression (Figure 7c). Additionally, knockdown of *PPARγ* or *p53* did not affect NaAR-induced polyUb-p62 expression (Supplementary Figure S9a,b). These results suggest that p53 and PPARγ act downstream of p62 and require its cooperation for their activities.

### 3.8. p62 Interacted with PPARγ and p53

**Figure 8:**
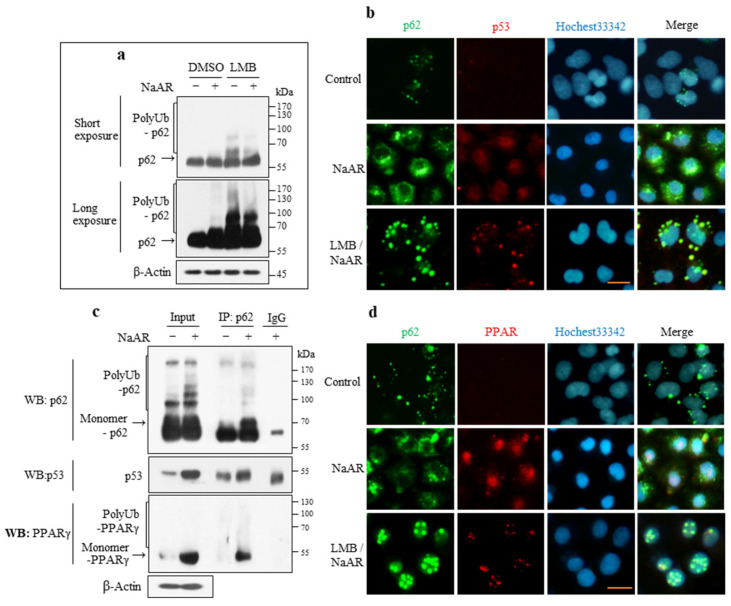
Subcellular localization of p62, p53, and PPARγ and their interactions in NaAR-exposed A549 cells. (**a**) Immunoblot of target proteins from cells treated with 65 μM NaAR for 18 h in the presence of either 25 nM LMB or DMSO. (**b**,**d**) Cells on coverslips were treated as in part (**a**), fixed, and stained with p62, p53, and PPARγ antibodies. Nuclei were counterstained with Hoechst 33342 (blue), and images were obtained by fluorescence microscopy. Scale bar: 25 µm. (**c**) Immunoblots of p62, p53, and PPARγ from cells treated with 65 µM NaAR for 12 h. After immunoblotting for p62 (input), 800 µg of the remaining protein was used for immunoprecipitation with p62 antibody, followed by immunoblotting for p62, p53, and PPARγ. β-Actin was used as the loading control (n = 3).

We further investigated the involvement of p62 in the molecular mechanism underlying PPARγ and p53 protein stability. The expression and subcellular locations of p62, p53, and PPARγ were examined in NaAR+LMB-treated cells. PolyUb-p62 accumulated significantly after LMB treatment (Figure 8a). Immunostaining of the control cells showed that p62 was localized as spots in the nucleus and cytoplasm, whereas p53 was not detected. In the NaAR-exposed cells, p62 appeared as prominent green spots aggregated in the cytoplasm close to the nuclear membrane, whereas red-stained p53 was detected at high intensity in the nucleus and low intensity in the cytoplasm. However, after LMB treatment, p62 and p53 were distributed as large globules in the cytoplasm and nucleus and completely merged (Figure 8b). In the control cells, PPARγ showed no significant staining. In the NaAR-exposed A549 cells, PPARγ was mainly present in the nucleus and in low levels in the cytoplasm. However, after LMB treatment, PPARγ decreased significantly in both compartments and was completely merged with p62 (Figure 8c). Further co-immunoprecipitation experiments showed that p62 interacted physically with PPARγ and p53 to form immunocomplexes (Figure 8d), indicating that it acts directly to stabilize these proteins.

## 4. Discussion

Toxic inorganic arsenic compounds induce multiple signaling pathways of cell death and survival by promoting oxidative stress, apoptosis, cell cycle arrest, and genotoxicity [31]. Dysregulation of these signaling pathways may contribute to carcinogenesis. Previous studies have shown that arsenic induces lung toxicity mainly via oxidative stress by depleting antioxidants and promoting mitochondrial dysfunction, leading to Bax- and caspase-dependent cell death [32,33]. Our present and previous studies showed that NaAR upregulated antioxidative enzymes, including superoxide dismutase 2 (SOD2) and HO-1, and disrupted the mitochondrial transmembrane potential in A549, H460, and H1299 cells [34,35]. Consistently, NaAR inhibited H460 cell growth by promoting apoptosis via the caspase-dependent and mitochondria-mediated apoptotic pathways [34]. H1299 cells showed high sensitivity to NaAR-induced parthanatos mediated by PARP-1 hyperactivation [35]. Although NaAR-exposed A549 cells did not show significant apoptotic changes at the morphological and biological levels, their proliferation was inhibited by the upregulation of cell cycle arrest-related proteins, such as p53 and p21. The downregulation of p53 and p21 due to PPARγ suppression indicates that cell cycle progression is positively regulated by PPARγ. Furthermore, PPARγ suppression induced necroptosis by reversing the NaAR-induced downregulation of MLKL expression, thereby increasing DNA damage and subsequent PARP-1 hyperactivation. Thus, the low sensitivity of A549 cells to NaAR is related to its high PPARγ expression.

The role of PPARγ as a lung cancer regulator through its inhibition of apoptosis, cell cycle arrest, invasion, and metastasis has emerged as a hot research topic [14]. We found that PPARγ was upregulated in response to NaAR in p53-expressing A549 and H460 cells, but not in p53-null H1299 cells, and its expression was positively correlated with p53 expression and negatively correlated with growth inhibition. As previously reported, arsenic-induced lung cytotoxicity was associated with parthanatos, BCL2/adenovirus E1B 19 kDa protein-interacting protein 3 (BNIP3L)/Nix-mediated endoplasmic reticulum stress, and autophagy in H1299 and H460 cells [34,35]. We thus reasoned that the induction of different signaling pathways in response to the same stimulus may be due to the PPARγ and p53 expression levels. Despite the important role of PPARγ in lung cancer, few studies have investigated its involvement in arsenic-induced lung carcinogenesis. In one study, cadmium and arsenic treatments of rat brain astrocytes enhanced PPARγ transcriptional activity and apoptosis, which were reduced by *PPARγ* knockdown or a PPARγ antagonist [36]. Therefore, the involvement of PPARγ in apoptosis in relation to arsenic-induced lung cancer warrants further study. Unlike normal human lung tissue, cancerous lung tissues express PPARγ [10], which exerts suppressive effects against the tumors by inhibiting cell proliferation and inducing apoptosis [13,14,15]. However, despite the fact that NaAR-exposed A549 cells expressed high PPARγ levels, they were the least sensitive to the toxin among the three cell lines studied. Although NaAR induced a significant decrease in procaspase-8 and an increase in RIP1 and RIP3, no significant degradation of procaspase-3 or PARP-1 was observed. Furthermore, the decrease in MLKL suggests that NaAR did not induce cell death via the caspase-dependent pathway, ripoptosis, or necroptosis. By contrast, PPARγ suppression resulted in PARP-1 hyperactivation and p-γH2AX and MLKL upregulation, indicating that DNA damage leads to PARP-1 hyperactivation and necroptosis. Although PARP-1 hyperactivation is not essential for necroptosis, we found that its induction via various experimental methods was positively correlated with the MLKL protein level. Consistent with this finding, Xu et al. [37] reported that PARP-1 activation induced RIP1–JNK-mediated necrosis. Moreover, RIP1-mediated PARP-1 activation is involved in TRAIL-induced necroptosis and oxidative stress-mediated necrosis [38,39]. Furthermore, typical DNA-damaging anticancer drugs such as etoposide and doxorubicin induced high p-γH2AX, p53, and p21 levels and PARP-1 cleavage in A549 cells but significantly decreased the PPARγ level. By contrast, cisplatin was insensitive to DNA damage and increased PPARγ (Supplementary Figure S10). Thus, these findings, along with those of previous studies, suggest that PPARγ may regulate PARP-1 activation to control cell survival or death.

Given its role as a transcription factor, PPARγ should be located in the nucleus [40], but studies have shown its distribution in the cytoplasm. Nuclear export signals in the DNA- and ligand-binding domains are recognized by nuclear export receptors, including CRM1 [41]. However, the molecular mechanism of nucleocytoplasmic PPARγ transport remains unclear. PPARγ binds MEK1 in response to phorbol esters and then translocates to the cytoplasm in a CRM1-dependent manner [42]. The cytoplasmic localization of PPARγ is either regulated by its phosphorylation by casein kinase-II and ERK or dependent on its ligands and Ca^2+^ concentration [41,43]. Since the nucleocytoplasmic transport of proteins is closely related to their functions, more studies are needed to elucidate the molecular mechanism of PPARγ subcellular translocation. Our fractionation studies showed that although PPARγ was mainly distributed in the nucleus, it also existed in the cytoplasm and particulate compartment and was degraded following LMB treatment. By contrast, p53, which is prominently present in the nucleus, accumulated in the cytoplasm after LMB treatment, suggesting that its cytoplasmic translocation may be partially independent of CRM1. However, inhibiting the proteasome resulted in the further accumulation of NaAR-induced PPARγ and partial restoration of its LMB-induced level reduction, but not of p53, suggesting that PPARγ protein stability may be regulated by proteasomes. Indeed, PPARγ was polyubiquitinated in an NaAR dose- and time-dependent manner. This is consistent with reports that PPARγ stability is regulated by the ubiquitin-proteasome pathway [44,45]. Thus, inhibiting CRM1 may have resulted in the deregulation of PPARγ, causing its degradation by the nuclear proteasome system and thereby releasing the physically attached p53 to freely translocate to the cytoplasm, leading to apoptosis. Nuclear p53 is involved in DNA repair, cell cycle arrest, and apoptosis, whereas cytoplasmic p53 plays a role in mitochondrial-dependent apoptosis [46,47]. In NaAR-exposed A549 cells, the uniformly distributed nuclear p53 molecules were redistributed as globular spots within the nucleus and cytoplasm following LMB treatment. This redistribution was accompanied by an increase in Bax and caspase-9 cleavage, suggesting a potential mitochondrial target for cytoplasmic p53. Indeed, we found that cytoplasmic p53 co-localized with the mitochondrial outer membrane protein TOMM20 in LMB-treated cells (Supplementary Figure S11). However, the mechanism that controls p53 translocation to the cytoplasm when PPARγ is downregulated remains unclear.

In contrast to cytoplasmic protein degradation, which is subject to various proteolytic systems including autophagy and proteasomes, nuclear protein degradation is predominantly proteasome-dependent [20,23]. p62 not only facilitates the delivery of Ub-cargo to proteasomes and autophagic clearance but also plays a critical role in protein quality control via nucleocytoplasmic trafficking [20]. In this study, polyUb-p62 accumulated in the nuclear, cytoplasmic, and particulate (containing autophagosomes) fractions of NaAR-exposed A549 cells and was distributed in a spot-like pattern. However, LMB treatment resulted in the further accumulation of polyUb-p62 aggregates in the nuclear and cytoplasmic compartments. Fu et al. [23,24] proposed that nuclear p62 aggregates (spherical liquid-like bodies) serve as active proteolytic sites for the proteasomal degradation of nuclear proteins, containing ubiquitinated targets, proteasomal subunits, and ubiquitin-conjugating and deubiquitinating enzymes. In the nucleus, p62 aggregates degrade the ubiquitin ligase ring finger protein 4 (RNF4), thereby stabilizing PML bodies and inhibiting their RNF4-mediated degradation by arsenic trioxide [24]. Upon its inhibition of nuclear protein export, p62 is required for polyUb-protein accumulation in PML bodies for proteasome degradation, thus aiding in nuclear protein quality control [22]. In this study, LMB resulted in the selective degradation of NaAR-induced PPARγ. Immunostaining of LMB-treated cells revealed that PPARγ was weakly stained because of its degradation and co-localization with p62 in the nucleus. Thus, we hypothesized that polyUb-PPARγ bound to the ubiquitin-binding domain of p62 after LMB treatment could be a target of PML bodies, which serve as a hub for post-transcriptional modifications. This process could promote polyUb-PPARγ degradation by the nuclear proteasome. Furthermore, the subcellular location and staining pattern of p53 after LMB treatment merged with those of p62 in both the nucleus and cytoplasm, and the two proteins interacted physically, indicating that p62 may be involved in the stabilization and intracellular trafficking of p53. Indeed, our previous studies and those of others have shown that p62 promotes cytoplasmic p53 translocation and induces apoptosis [34,48]. Taken together, these results suggest that PPARγ inhibits apoptosis by inhibiting cytoplasmic p53 translocation.

In the extrinsic pathway of death receptor-mediated apoptosis, activation of death receptors, including Fas and tumor necrosis factor receptor 1 (TNFR1), leads to the recruitment of FADD, which subsequently recruits caspase-8 to form the death-inducing signaling complex (DISC). This process results in caspase-8 activation and subsequent activation of downstream caspases, ultimately leading to PARP-1 cleavage [49]. RIP1, a serine/threonine kinase, plays a critical role in regulating cell death and survival depending on the cellular context [50]. Although RIP1 is not a primary component of Fas signaling, it can be recruited via an interaction with FADD, where its K63-linked ubiquitination promotes cell survival through NF-κB activation. In contrast, deubiquitinated RIP1 forms either complex IIa (RIP1–FADD–caspase-8) or complex IIb (RIP1–RIP3–MLKL), depending on caspase-8 activity, thereby inducing apoptosis or necroptosis [51]. In the present study, RIP1 was degraded at low NaAR concentrations but became ubiquitinated and upregulated at concentrations above 45 μM, suggesting stabilization within complex I to enhance cell survival. Consistently, increased K63-linked RIP1 ubiquitination and elevated NF-κB p65 levels were observed. Furthermore, NaAR exposure reduced caspase-8 and MLKL expression without activating downstream caspases, indicating suppression of RIP1-mediated apoptotic signaling. Although recent studies have linked arsenic-induced necroptosis to myocardial inflammation in vivo [52], the role of arsenic in regulating necroptosis or ripoptosis remains insufficiently explored.

Notably, accumulating evidence suggests that the biological effects of arsenic are highly dependent on its chemical form. For example, Rainey et al. [53] reported that sodium arsenite and arsenic trioxide differentially modulate oxidative stress responses in lymphoblastoid cells through intricate crosstalk among mitochondria, autophagy, and cell death pathways. In line with these observations, our findings demonstrate that sodium arsenite specifically regulates PPARγ-dependent signaling, thereby influencing the balance between apoptosis and necroptosis in NSCLC cells. These results underscore the importance of arsenic speciation in determining cellular responses and downstream molecular mechanisms.

However, several limitations should be considered. Our findings are based on in vitro models, which may not fully recapitulate in vivo conditions. Moreover, we did not directly compare the effects of trivalent arsenic compounds (As(III)) with arsenate (As(V)), which may differ in their biological mechanisms, with As(V) generally being regarded as less toxic than As(III). Future studies incorporating in vivo validation and more comprehensive mechanistic analyses will be necessary to further elucidate the systemic and context-dependent effects of arsenic.

## 5. Conclusions

**Figure 9:**
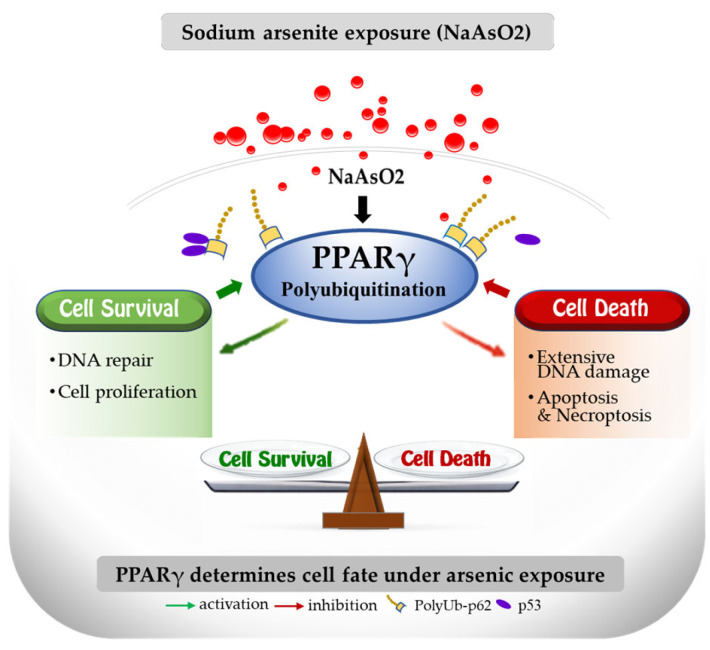
Sodium arsenite (NaAR) induces polyubiquitination of PPARγ, which promotes DNA damage responses while suppressing apoptosis and necroptosis through NF-κB activation and MLKL downregulation. Disruption of PPARγ or modulation of PARP-1 activity shifts the balance toward necroptosis or apoptosis. p53 and p62 cooperate with PPARγ to regulate cell fate in A549 cells.

Our findings indicate that PPARγ plays a critical role in NaAR-driven lung carcinogenesis. This effect appears to involve activation of DNA damage response pathways along with suppression of both apoptosis and necroptosis under the regulation of PPARγ. In addition, the functional activity of PPARγ depends on its interaction with p62 and p53. Collectively, these results suggest that PPARγ may serve as a promising therapeutic target for enhancing the responsiveness of NSCLC to NaAR, while also providing insights into mechanisms underlying anticancer drug resistance. A graphical abstract is included to summarize the proposed mechanisms and illustrate the balance between cell survival and cell death pathways (Figure 9).

## Data Availability

The original contributions presented in this study are included in the article/Supplementary Materials. Further inquiries can be directed to the corresponding author.

## Supplementary Materials

The following supporting information can be downloaded at: https://www.mdpi.com/article/10.3390/cells15080659/s1. Figure S1: Morphological changes in sodium arsenite (NaAR)-exposed H1299 cells; Figure S2: Expression of apoptosis-, necroptosis-, and DNA damage response-related proteins following NaAR incubation over time; Figure S3: Expression of necroptosis- and DNA damage-related proteins in NaAR-treated H1299 cells; Figure S4: K48- and K63-linked polyubiquitination in NaAR-treated H1299 cells; Figure S5: AKT inhibition blocked NaAR-induced p-ERK and PPARγ and induced PARP-1 cleavage; Figure S6: p53 induces DNA damage, leading to PARP-1 hyperac-tivation and necroptosis through PPARγ regulation; Figure S7: PPARγ protein stability is regulated by proteasomal activity. Figure S8: Nuclear proteasome-mediated degradation of PPARγ; Figure S9: p53 and PPARγ act downstream of p62; Figure S10: PPARγ may control cell survival or death via PARP-1; Figure S11: Cytoplasmic p53 contributes to mitochondrial-mediated apoptosis.
