# Supplementary material for: Arsenic-Induced PPARγ, with the Coordinated Action of p62, Inhibits Apoptosis and Necroptosis and Activates the DNA Damage Response in A549 Lung Cancer Cells, Leading to Carcinogenesis
Source: Cells. 2026 Apr 8;15(8):659. doi: 10.3390/cells15080659 (PMC13115141; doi:10.3390/cells15080659)
Supplement: Supplementary file 1 [file cells-15-00659-s001.zip › cells-4230290-supplementary.pptx]

## Slide 1
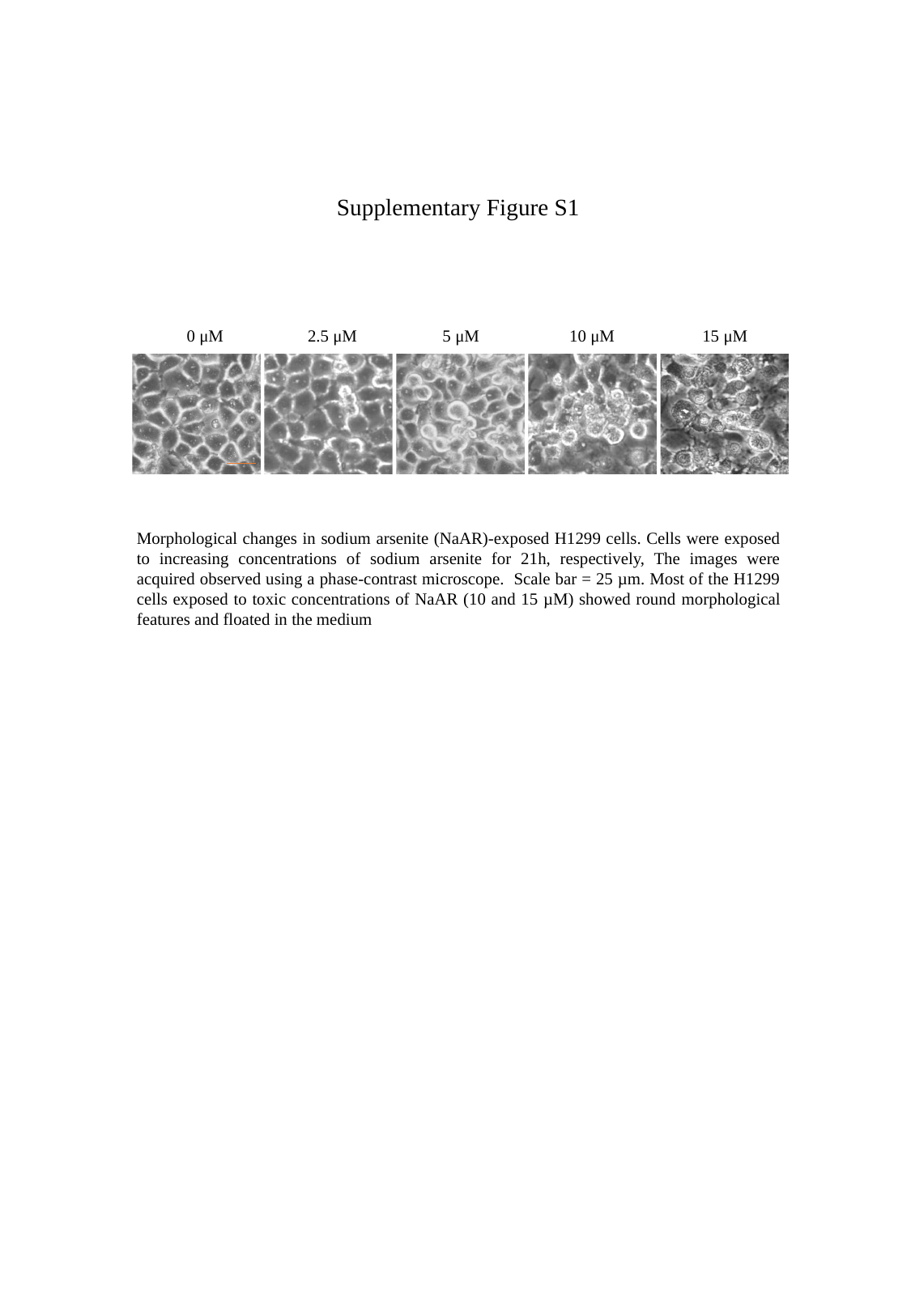

Supplementary Figure S1
0 μM
2.5 μM
5 μM
10 μM
15 μM
Morphological changes in sodium arsenite (NaAR)-exposed H1299 cells. Cells were exposed to increasing concentrations of sodium arsenite for 21h, respectively, The images were acquired observed using a phase-contrast microscope. Scale bar = 25 µm. Most of the H1299 cells exposed to toxic concentrations of NaAR (10 and 15 µM) showed round morphological features and floated in the medium

## Slide 2
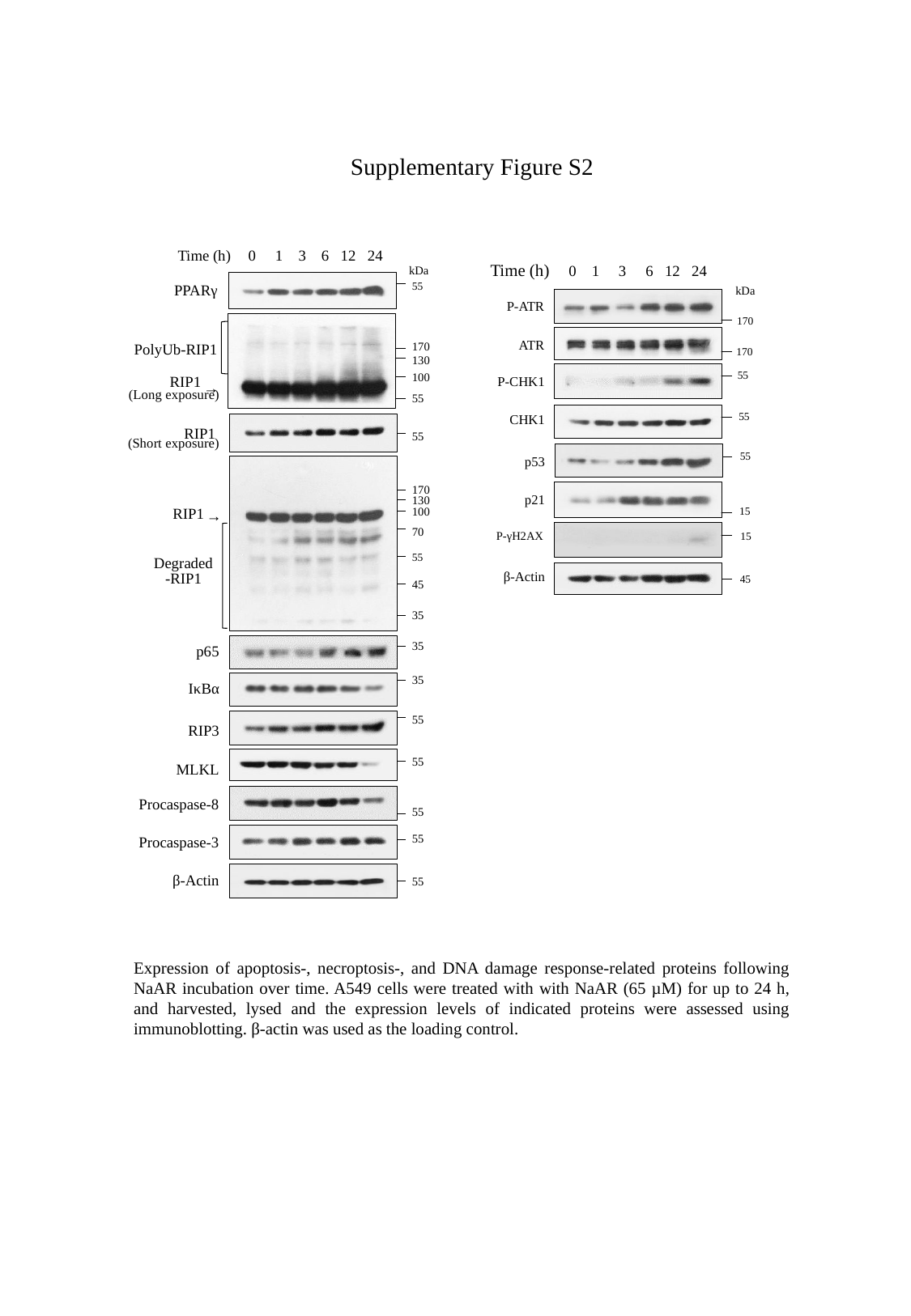

Supplementary Figure S2
0 1 3 6 12 24
Time (h)
Time (h)
0 1 3 6 12 24
kDa
55
PPARγ
kDa
P-ATR
170
ATR
170
PolyUb-RIP1
170
130
→
55
100
RIP1
P-CHK1
(Long exposure)
55
55
CHK1
RIP1
55
(Short exposure)
55
p53
170
→
p21
130
15
RIP1
100
70
P-γH2AX
15
55
Degraded
 -RIP1
β-Actin
45
45
35
35
p65
35
IκBα
55
RIP3
55
MLKL
Procaspase-8
55
55
Procaspase-3
β-Actin
55
Expression of apoptosis-, necroptosis-, and DNA damage response-related proteins following NaAR incubation over time. A549 cells were treated with with NaAR (65 µM) for up to 24 h, and harvested, lysed and the expression levels of indicated proteins were assessed using immunoblotting. β-actin was used as the loading control.

## Slide 3
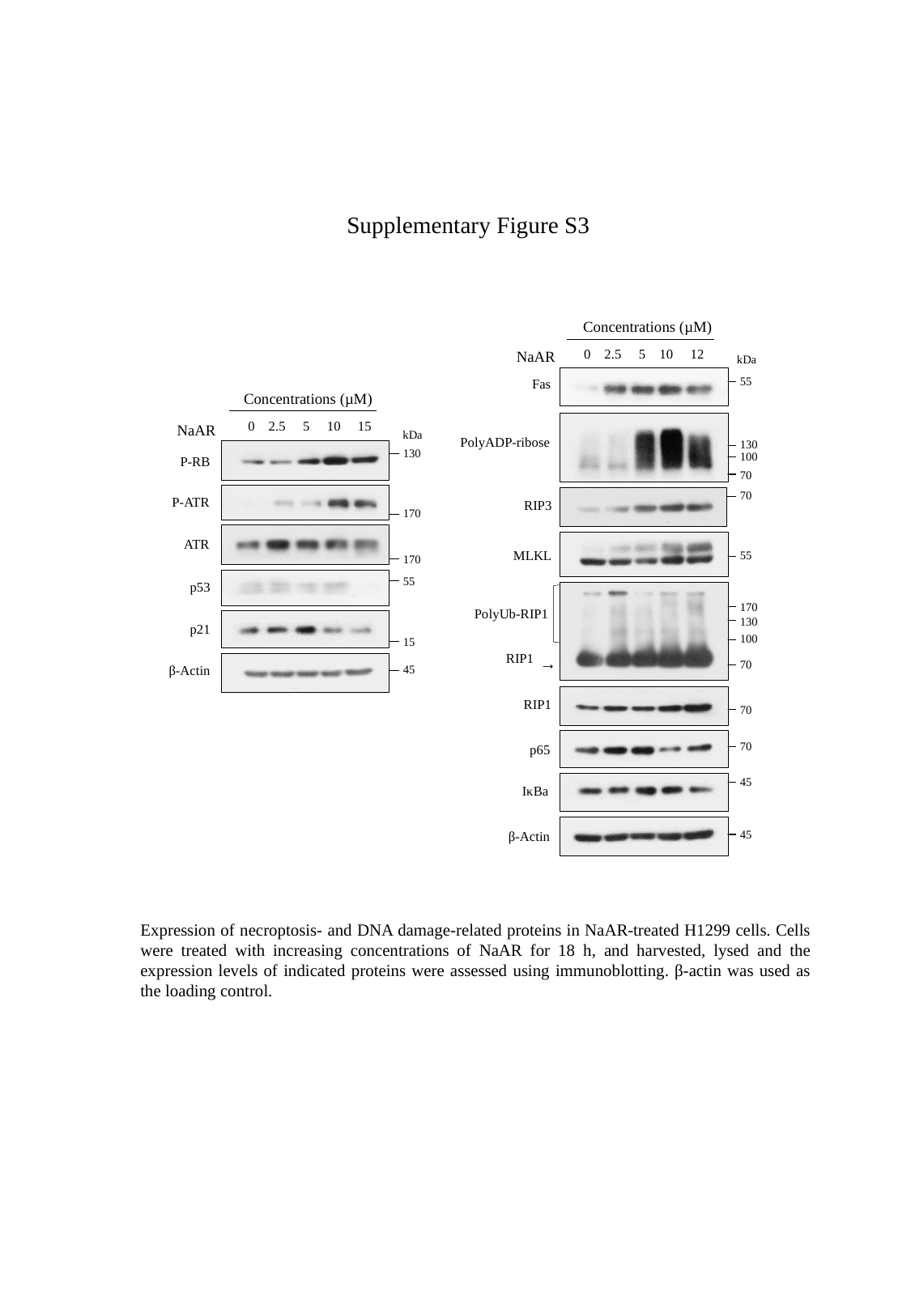

Supplementary Figure S3
Concentrations (µM)
0 2.5 5 10 12
NaAR
kDa
55
Fas
Concentrations (µM)
0 2.5 5 10 15
NaAR
kDa
PolyADP-ribose
130
130
100
P-RB
70
70
P-ATR
RIP3
170
ATR
MLKL
55
170
55
p53
170
PolyUb-RIP1
130
p21
100
→
15
RIP1
70
β-Actin
45
RIP1
70
70
p65
45
IκBa
45
β-Actin
Expression of necroptosis- and DNA damage-related proteins in NaAR-treated H1299 cells. Cells were treated with increasing concentrations of NaAR for 18 h, and harvested, lysed and the expression levels of indicated proteins were assessed using immunoblotting. β-actin was used as the loading control.

## Slide 4
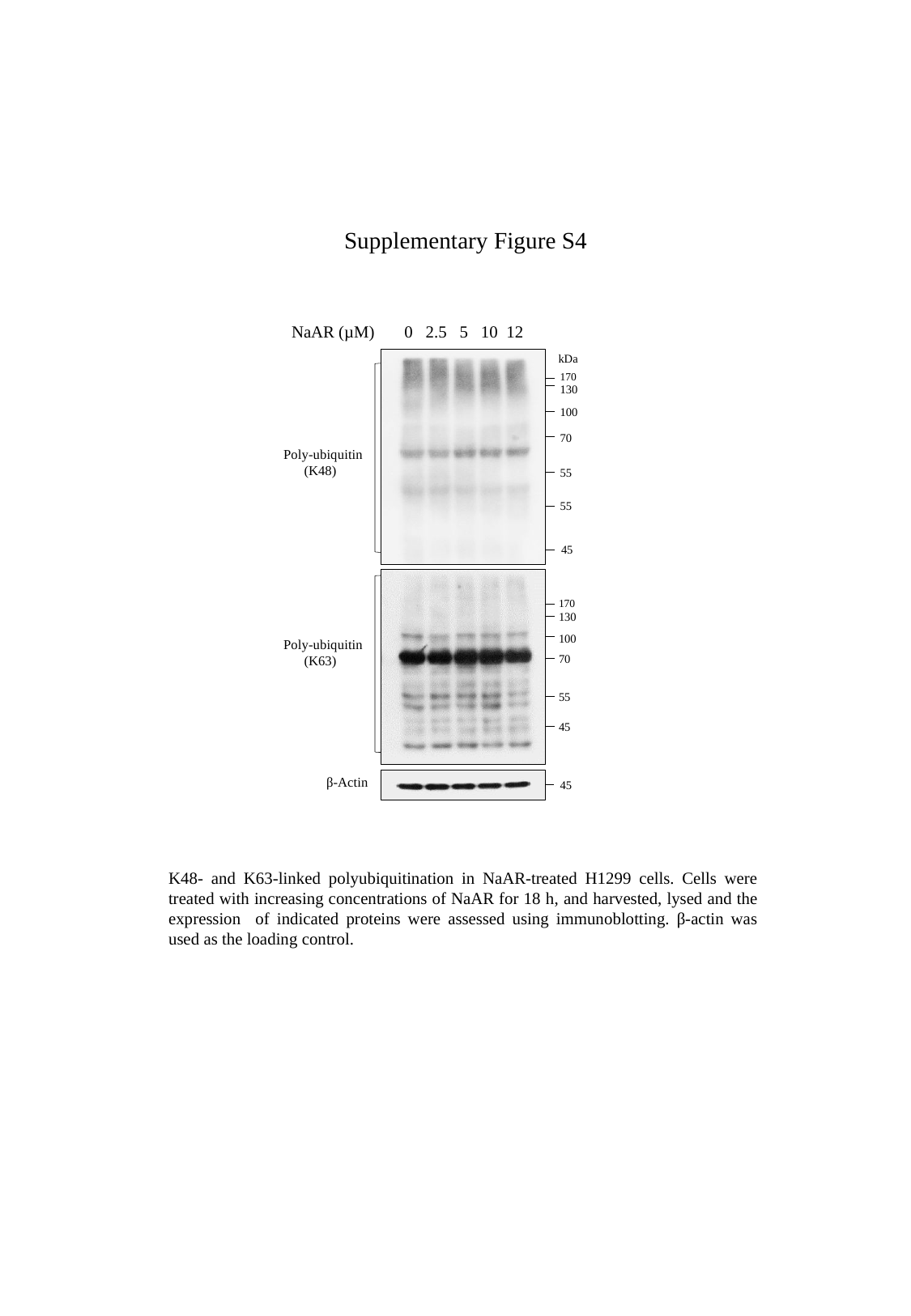

Supplementary Figure S4
 NaAR (µM) 0 2.5 5 10 12
Poly-ubiquitin
 (K48)
kDa
170
130
100
70
55
55
45
170
130
100
Poly-ubiquitin
 (K63)
70
55
45
β-Actin
45
K48- and K63-linked polyubiquitination in NaAR-treated H1299 cells. Cells were treated with increasing concentrations of NaAR for 18 h, and harvested, lysed and the expression of indicated proteins were assessed using immunoblotting. β-actin was used as the loading control.

## Slide 5
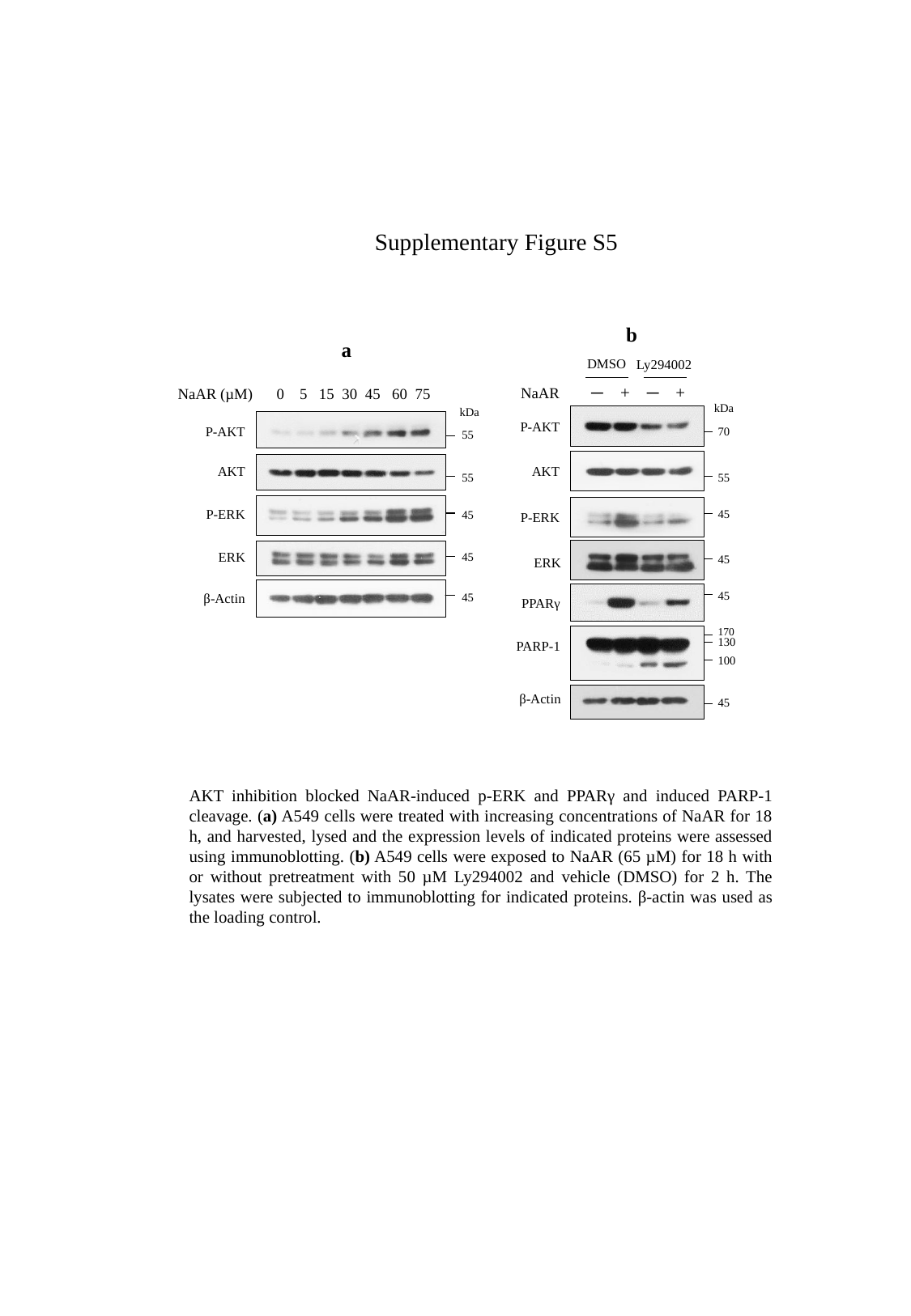

Supplementary Figure S5
b
a
DMSO
Ly294002
─ + ─ +
 NaAR
 NaAR (µM)
0 5 15 30 45 60 75
kDa
kDa
P-AKT
P-AKT
70
55
AKT
AKT
55
55
P-ERK
45
45
P-ERK
ERK
45
45
ERK
45
β-Actin
45
PPARγ
170
β-Actin
130
PARP-1
100
45
AKT inhibition blocked NaAR-induced p-ERK and PPARγ and induced PARP-1 cleavage. (a) A549 cells were treated with increasing concentrations of NaAR for 18 h, and harvested, lysed and the expression levels of indicated proteins were assessed using immunoblotting. (b) A549 cells were exposed to NaAR (65 µM) for 18 h with or without pretreatment with 50 µM Ly294002 and vehicle (DMSO) for 2 h. The lysates were subjected to immunoblotting for indicated proteins. β-actin was used as the loading control.

## Slide 6
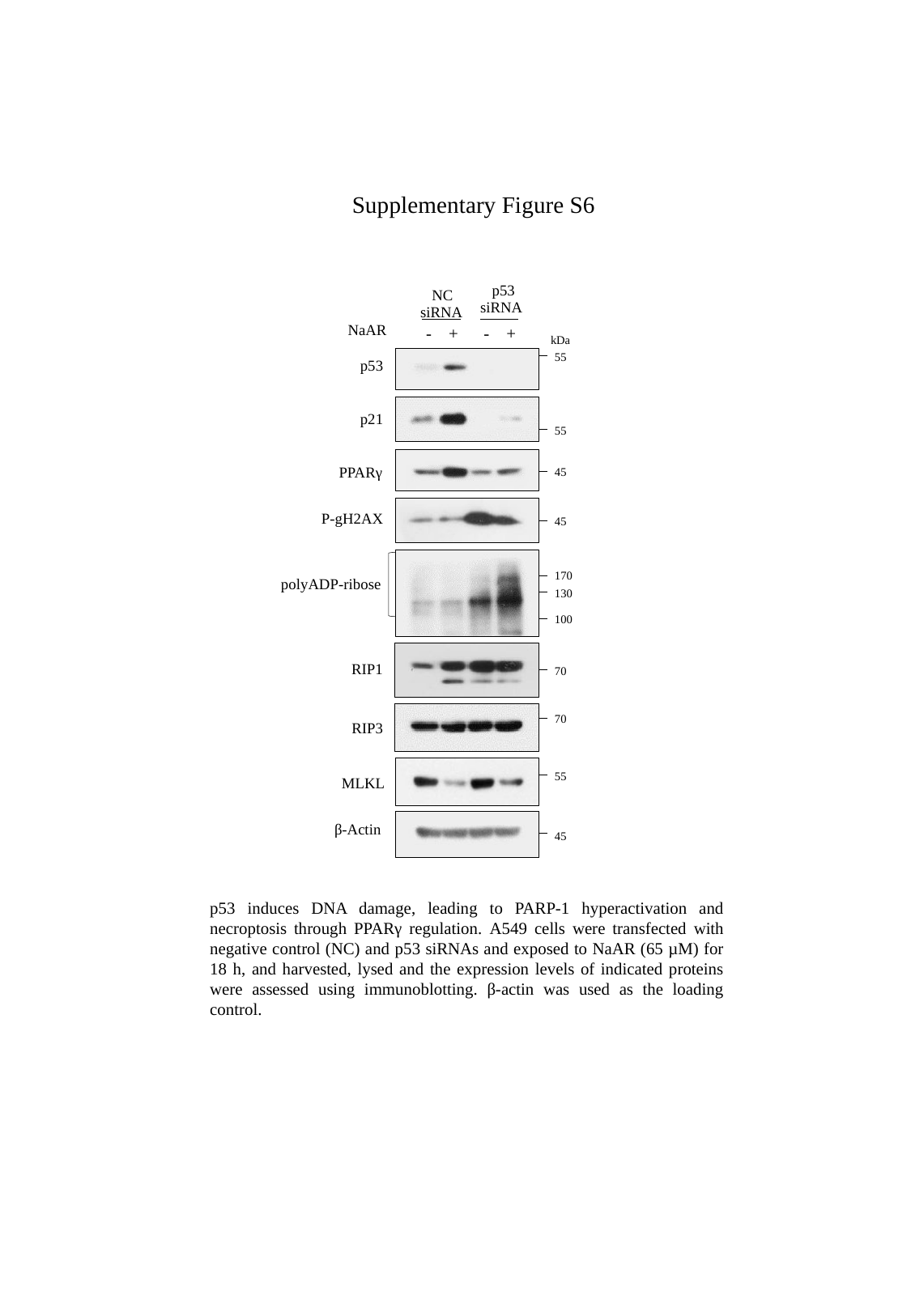

Supplementary Figure S6
 p53
siRNA
 NC
siRNA
 NaAR
- + - +
kDa
55
p53
p21
55
PPARγ
45
P-gH2AX
45
170
polyADP-ribose
130
100
RIP1
70
70
RIP3
55
MLKL
β-Actin
45
p53 induces DNA damage, leading to PARP-1 hyperactivation and necroptosis through PPARγ regulation. A549 cells were transfected with negative control (NC) and p53 siRNAs and exposed to NaAR (65 µM) for 18 h, and harvested, lysed and the expression levels of indicated proteins were assessed using immunoblotting. β-actin was used as the loading control.

## Slide 7
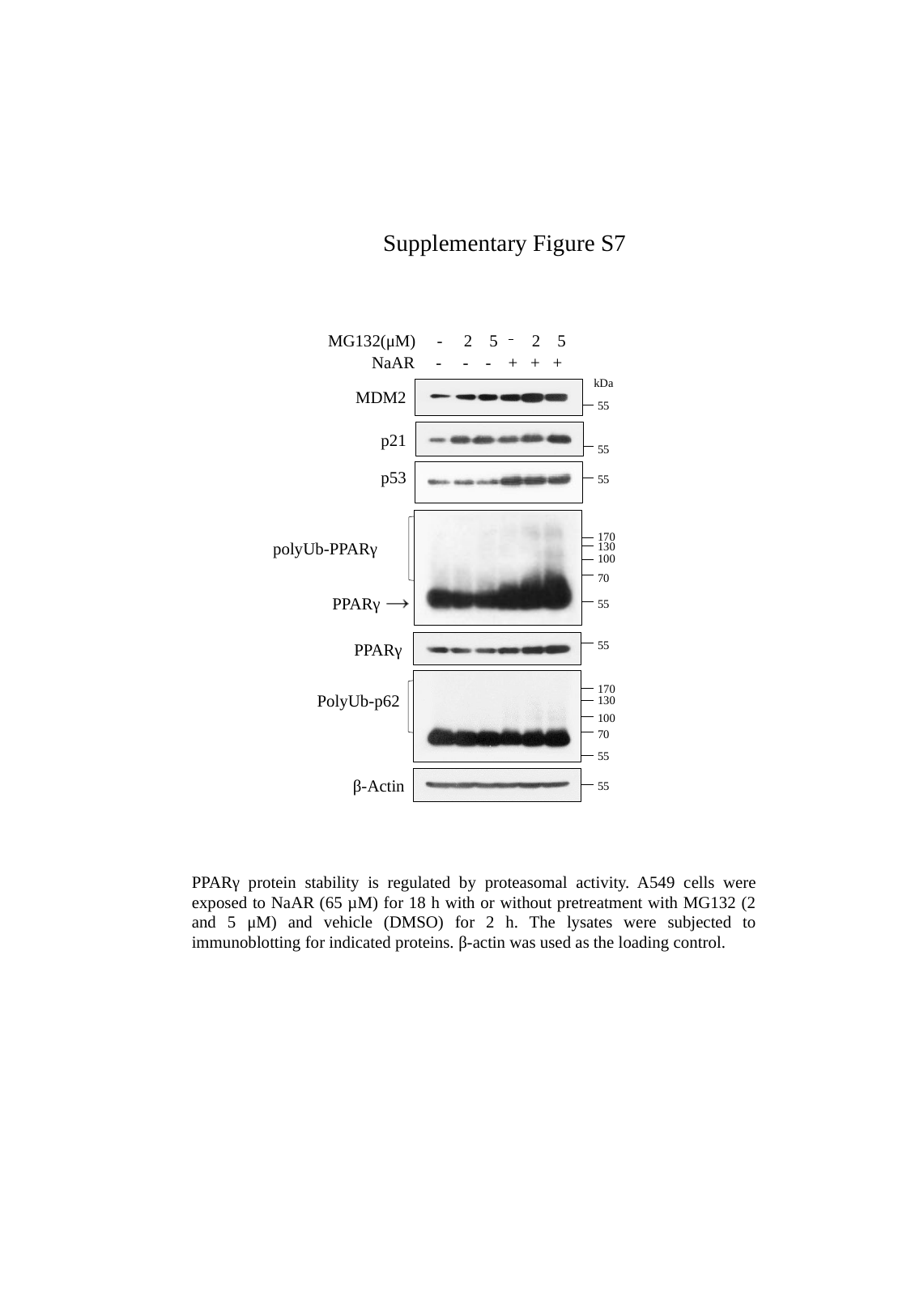

Supplementary Figure S7
 MG132(μM) - 2 5 2 5
MDM2
p21
p53
polyUb-PPARγ
PPARγ
PPARγ
PolyUb-p62
β-Actin
NaAR - - - + + +
kDa
55
55
55
170
130
100
70
55
55
170
130
100
70
55
55
PPARγ protein stability is regulated by proteasomal activity. A549 cells were exposed to NaAR (65 µM) for 18 h with or without pretreatment with MG132 (2 and 5 μM) and vehicle (DMSO) for 2 h. The lysates were subjected to immunoblotting for indicated proteins. β-actin was used as the loading control.

## Slide 8
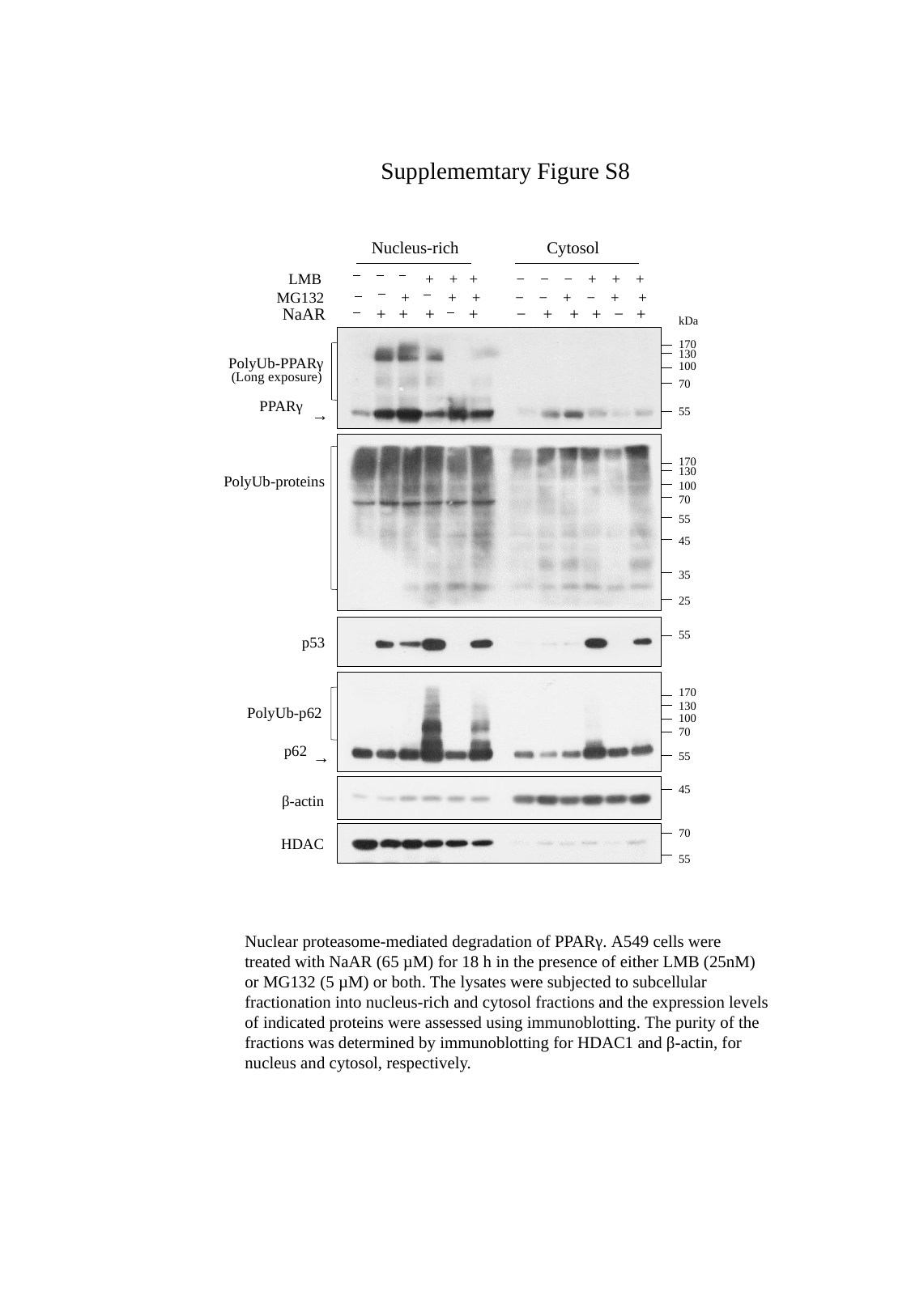

Supplememtary Figure S8
Nucleus-rich
Cytosol
 PolyUb-PPARγ
 (Long exposure)
→
 PPARγ
PolyUb-proteins
p53
PolyUb-p62
→
p62
β-actin
 HDAC
 LMB + + + − − − + + +
MG132 + + + − − + − + +
NaAR + + + + − + + + − +
kDa
170
130
100
70
55
170
130
100
70
55
45
35
25
55
170
130
100
70
55
45
70
55
Nuclear proteasome-mediated degradation of PPARγ. A549 cells were treated with NaAR (65 µM) for 18 h in the presence of either LMB (25nM) or MG132 (5 µM) or both. The lysates were subjected to subcellular fractionation into nucleus-rich and cytosol fractions and the expression levels of indicated proteins were assessed using immunoblotting. The purity of the fractions was determined by immunoblotting for HDAC1 and β-actin, for nucleus and cytosol, respectively.

## Slide 9
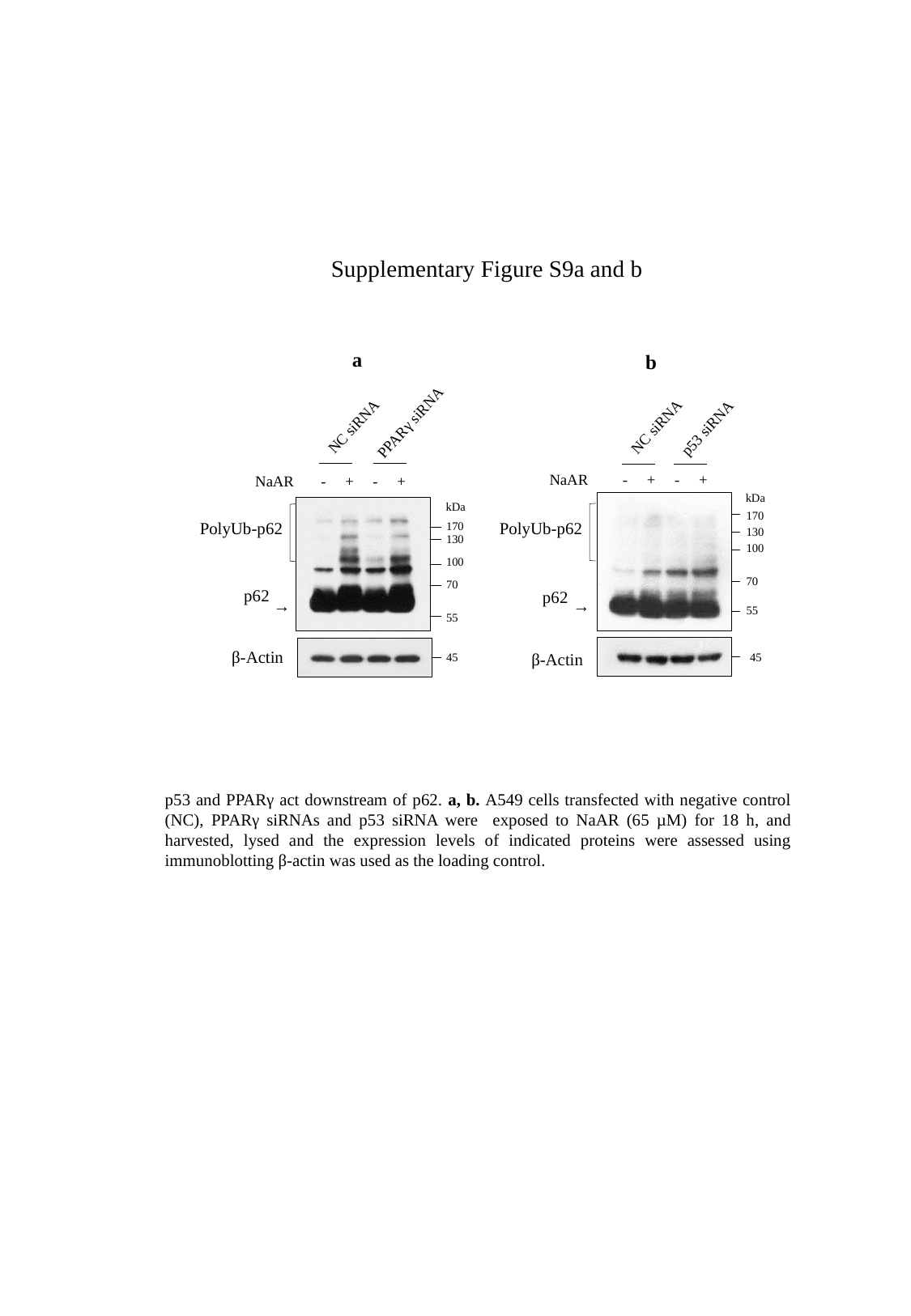

Supplementary Figure S9a and b
a
b
p53 siRNA
NC siRNA
 PolyUb-p62
→
 p62
β-Actin
170
130
100
70
55
45
PPARγ siRNA
NC siRNA
NaAR - + - +
NaAR - + - +
kDa
kDa
170
 PolyUb-p62
130
100
→
70
 p62
55
β-Actin
45
p53 and PPARγ act downstream of p62. a, b. A549 cells transfected with negative control (NC), PPARγ siRNAs and p53 siRNA were exposed to NaAR (65 µM) for 18 h, and harvested, lysed and the expression levels of indicated proteins were assessed using immunoblotting β-actin was used as the loading control.

## Slide 10
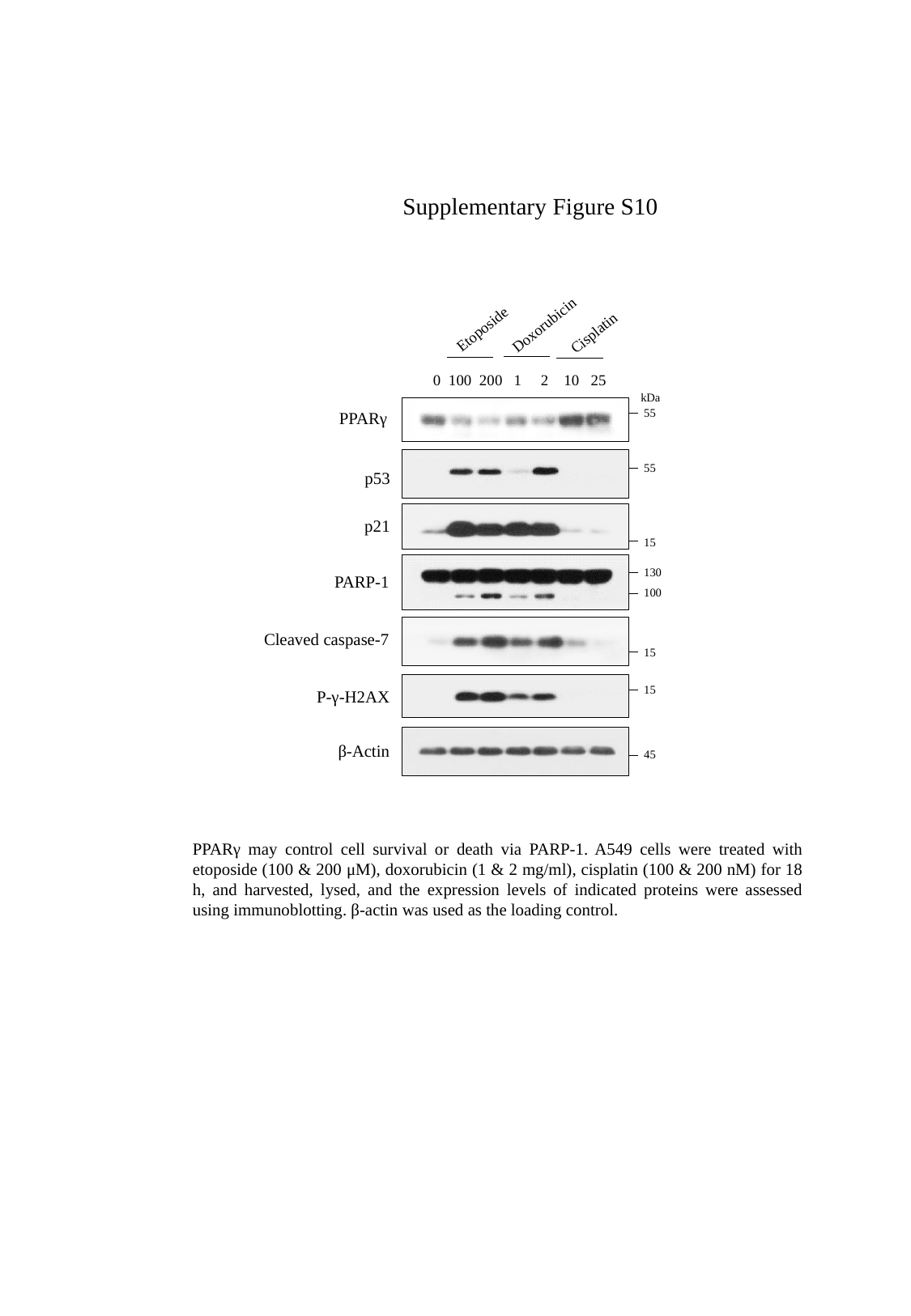

Supplementary Figure S10
Doxorubicin
Etoposide
Cisplatin
0 100 200 1 2 10 25
 PPARγ
p53
p21
PARP-1
Cleaved caspase-7
P-γ-H2AX
β-Actin
kDa
55
55
15
130
100
15
15
45
PPARγ may control cell survival or death via PARP-1. A549 cells were treated with etoposide (100 & 200 μM), doxorubicin (1 & 2 mg/ml), cisplatin (100 & 200 nM) for 18 h, and harvested, lysed, and the expression levels of indicated proteins were assessed using immunoblotting. β-actin was used as the loading control.

## Slide 11
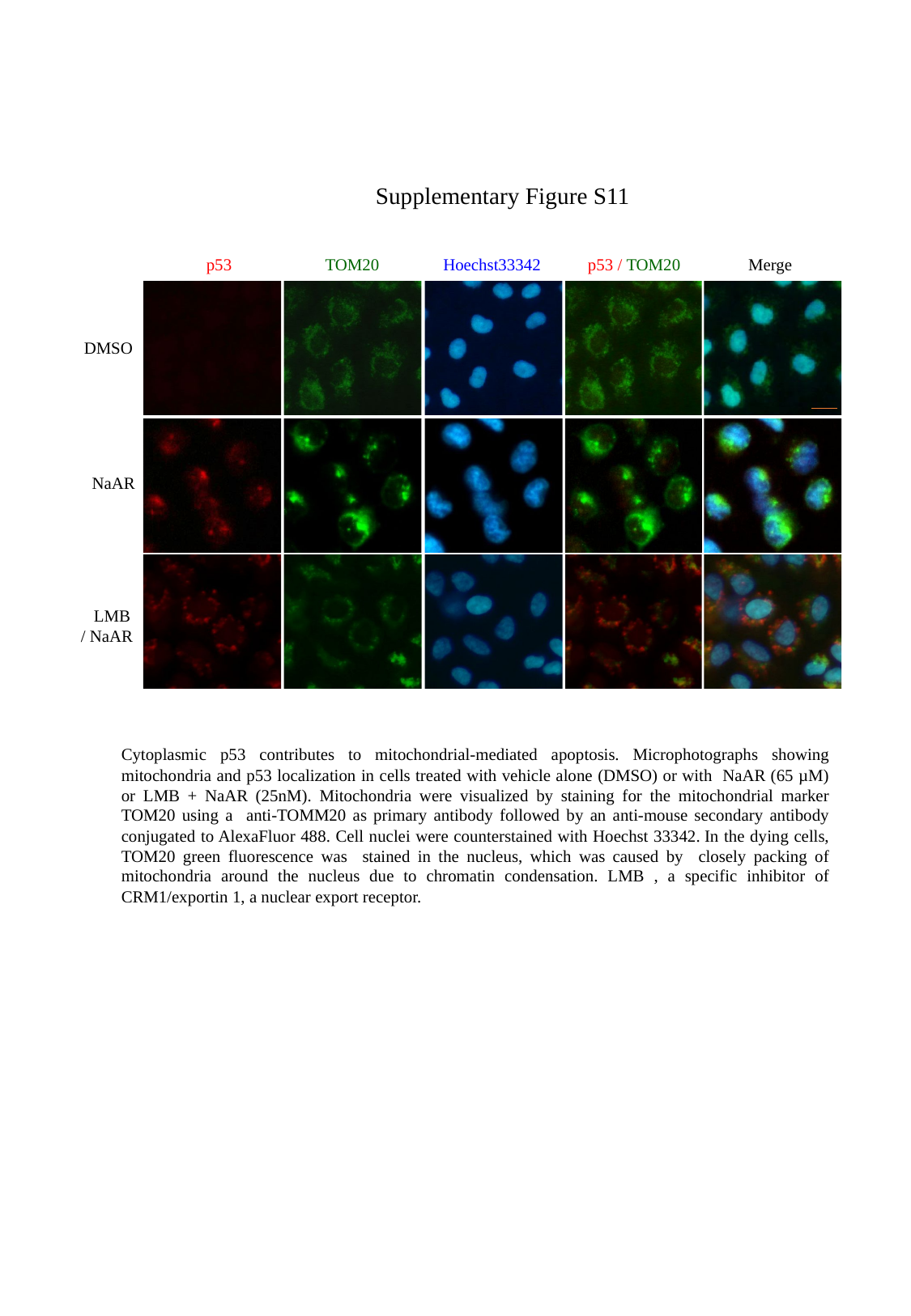

Supplementary Figure S11
p53 TOM20 Hoechst33342 p53 / TOM20 Merge
DMSO
NaAR
 LMB
/ NaAR
Cytoplasmic p53 contributes to mitochondrial-mediated apoptosis. Microphotographs showing mitochondria and p53 localization in cells treated with vehicle alone (DMSO) or with NaAR (65 µM) or LMB + NaAR (25nM). Mitochondria were visualized by staining for the mitochondrial marker TOM20 using a anti-TOMM20 as primary antibody followed by an anti-mouse secondary antibody conjugated to AlexaFluor 488. Cell nuclei were counterstained with Hoechst 33342. In the dying cells, TOM20 green fluorescence was stained in the nucleus, which was caused by closely packing of mitochondria around the nucleus due to chromatin condensation. LMB , a specific inhibitor of CRM1/exportin 1, a nuclear export receptor.
